# Supplementary material for: Upregulation of long non-coding RNA SNHG16 promotes diabetes-related RMEC dysfunction via activating NF-κB and PI3K/AKT pathways
Source: Mol Ther Nucleic Acids. 2021 Feb 4;24:512–27. doi: 10.1016/j.omtn.2021.01.035 (PMC8056184; doi:10.1016/j.omtn.2021.01.035)
Supplement: Document S1. Figures S1–S9, Tables S1 and S2, and Supplemental sequence [file mmc1.pdf]

OMTN, Volume *24*

Supplemental information

Upregulation of long non-coding RNA SNHG16 promotes diabetes-related RMEC dysfunction via activating NF-κB and PI3K/AKT pathways

Fei Cai, Huanzong Jiang, Yan Li, Qin Li, and Chao Yang

Figure S1 HG enhances hRMEC viability and proliferation. (A) CCK-8 assay was applied to evaluate the cell viability after treated with HG at 0h, 12h and 48h. N=3 in each group. (B) Cell proliferation ratio of each group (0h, 12h and 48h) was illustrated through calculating the percentage of EdU positive cells using EdU assay. N=3 in each group. (C) The morphology of hRMECs treated with LG or HG was observed under a microscope. (D) The ROS level in HG-treated hRMECs after SNHG16 silencing. (E) Apoptosis-related proteins were detected in hRMECs after indicated transfections. (F) The levels of HIF-1α and VEGF were measured in hRMECs after indicated transfections. All data were acquired from three independent experiments and presented as the mean ± SD. *p < 0.05, **p < 0.01.

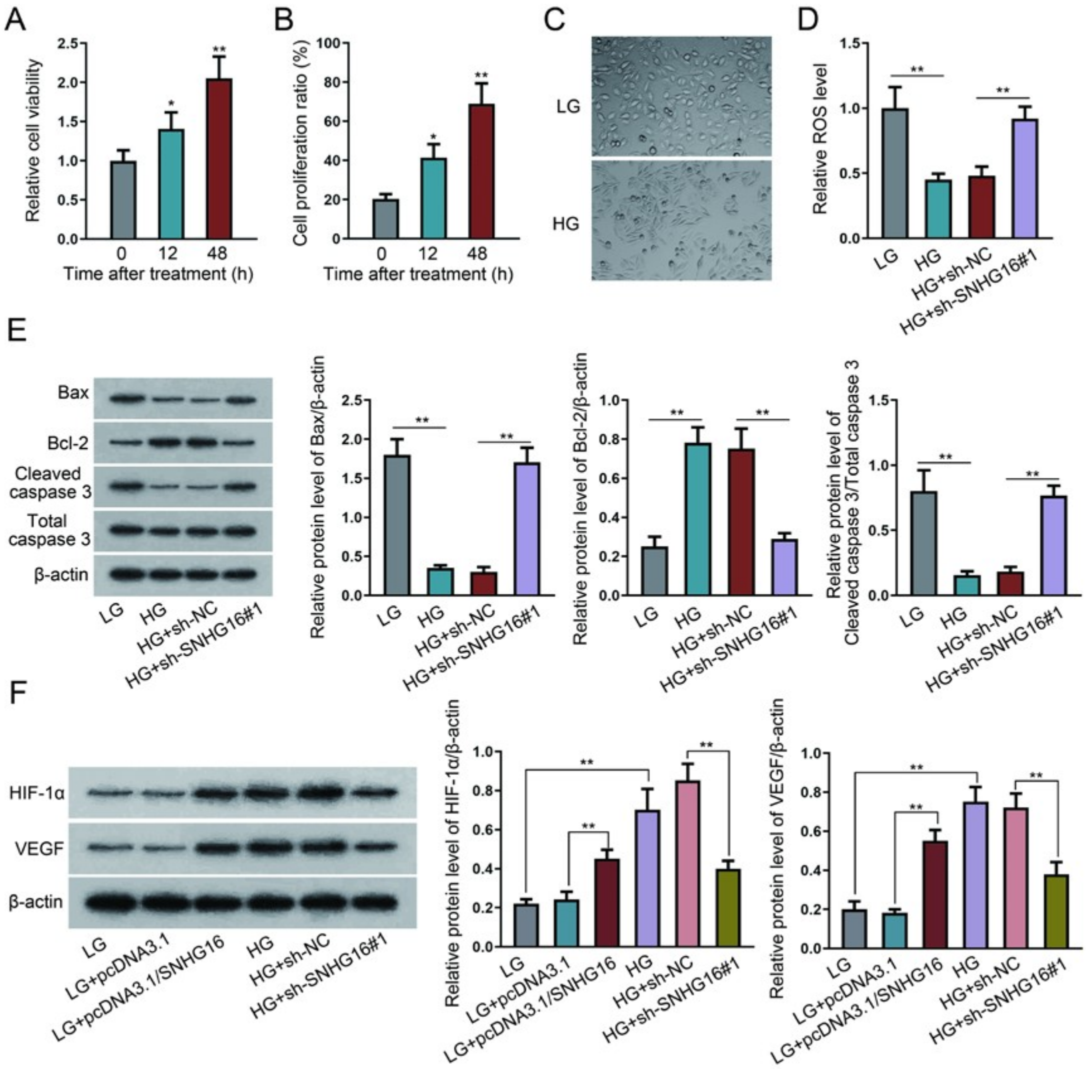

A
Relative cell viability
Time after treatment (h)
B
Cell proliferation ratio (%)
Time after treatment (h)
C
LG
HG
D
Relative ROS level
LG
HG
HG+sh-NC
HG+sh-SNHG16#1
E
Bax
Bcl-2
Cleaved caspase 3
Total caspase 3
β-actin
Relative protein level of Bax/β-actin
Relative protein level of Bcl-2/β-actin
Relative protein level of Cleaved caspase 3/Total caspase 3
F
HIF-1α
VEGF
β-actin
LG
LG+pcDNA3.1
LG+pcDNA3.1/SNHG16
HG
HG+sh-NC
HG+sh-SNHG16#1
Relative protein level of HIF-1α/β-actin
Relative protein level of VEGF/β-actin

Figure S2 The role of the snoRNAs encoded by SNHG16 in modulating hRMEC functions. (A) The expression level of different isoforms of SNHG16 in hRMECs cultured under HG or LG condition for 48 h was examined by qRT-PCR. N=3 in each group. (B) The expression levels of three snoRNAs were examined in hRMECs cultured under HG or LG condition for 48 h was examined by qRT-PCR. N=3 in each group. (C) The expression levels of three snoRNAs were examined in LG-induced hRMEC with SNHG16 overexpression or HG-induced hRMEC with SNHG16 silencing by qRT-PCR. N=3 in each group. (D) The cell viability was measured by CCK-8 assay after silencing of three snoRNAs. N=3 in each group. (E) EdU assay was applied to detect proliferative cells in HG-induced hRMEC after knockdown of three snoRNAs. N=3 in each group. (F) Wound healing assay revealed the snoRNAs silencing on hRMEC migration. N=3 in each group. (G) Migrated cell number of HG-induced hRMEC was counted using Transwell assay after silencing of snoRNAs. N=3 in each group. (H) qRT-PCR analysis of VEGF mRNA level in each group. N=3 in each group. (I) ELISA showed VEGF protein level influenced by snoRNAs knockdown. N=3 in each group. (J) The effect of snoRNAs knockdown on tube formation ability was evaluated by tube formation assay. N=3 in each group. All data were acquired from three independent experiments and presented as the mean ± SD.

**$p < 0.01$, n.s. indicated difference was not statistically significant.

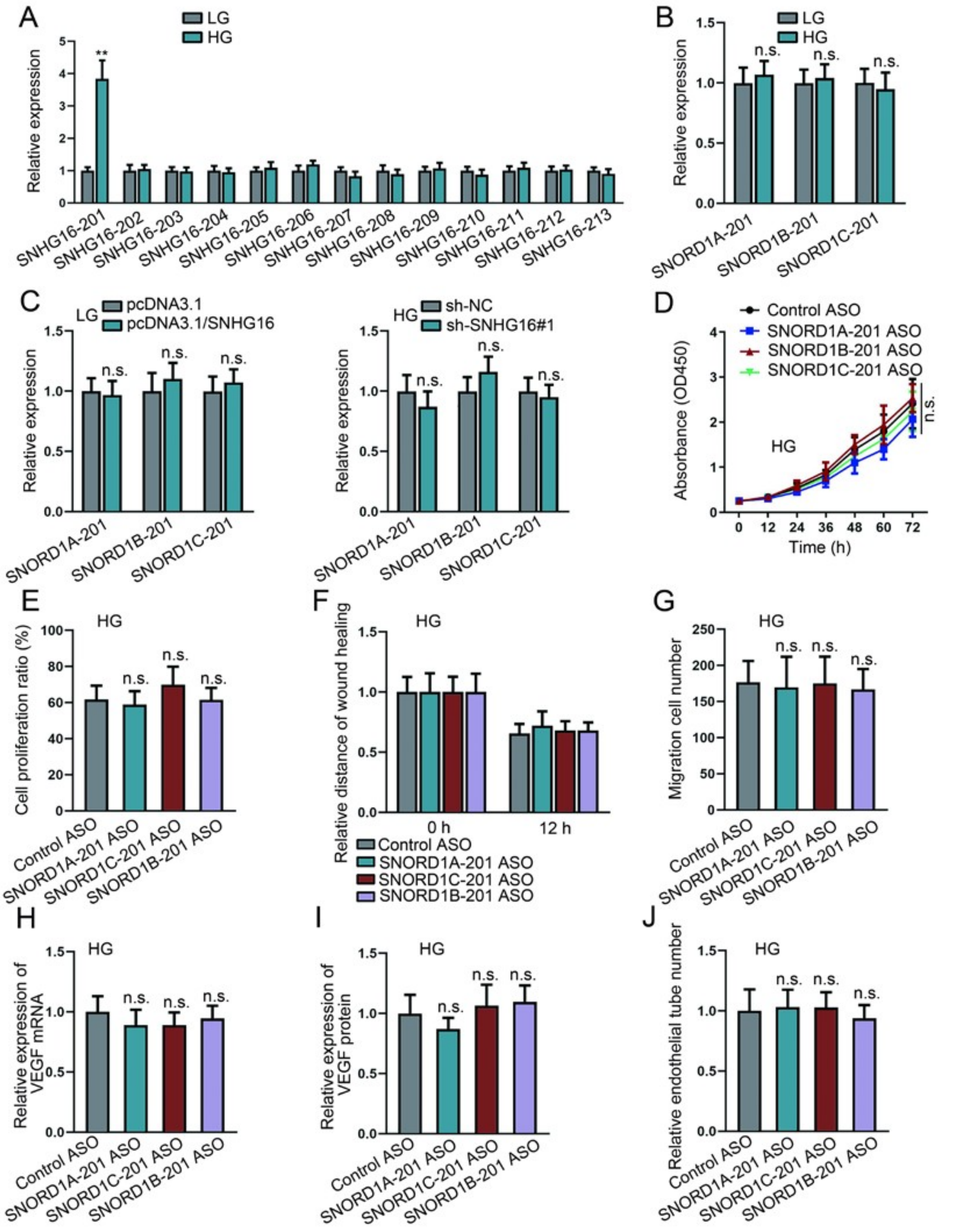

A
LG
HG
Relative expression
**
SNHG16-201
SNHG16-202
SNHG16-203
SNHG16-204
SNHG16-205
SNHG16-206
SNHG16-207
SNHG16-208
SNHG16-209
SNHG16-210
SNHG16-211
SNHG16-212
SNHG16-213
B
n.s.
SNORD1A-201
SNORD1B-201
SNORD1C-201
C
pcDNA3.1
pcDNA3.1/SNHG16
sh-NC
sh-SNHG16#1
D
Control ASO
SNORD1A-201 ASO
SNORD1B-201 ASO
SNORD1C-201 ASO
Absorbance (OD450)
Time (h)
E
Cell proliferation ratio (%)
SNORD1A-201 ASO
SNORD1C-201 ASO
SNORD1B-201 ASO
F
Relative distance of wound healing
0 h
12 h
G
Migration cell number
H
Relative expression of VEGF mRNA
I
Relative expression of VEGF protein
J
Relative endothelial tube number

Figure S3 The effect of SNHG16 on the expression of IRAK1 and IRS1. Experiments were conducted in cells treated with LG (5 mmol/L) or HG (25 mmol/L) for 48 hours. (A and B) IRAK1 and IRS1 expression in hRMECs cultured with HG, LG or osmotic control was detected using qRT-PCR and Western blot analyses. N=3 in each group. (C and D) qRT-PCR and Western blot analyses showed the mRNA and protein levels of IRAK1 and IRS1 influenced by SNHG16 overexpression or knockdown. N=3 in each group. All data were acquired from three independent experiments and presented as the mean ± SD. **p < 0.01, n.s. indicated difference was not statistically significant.

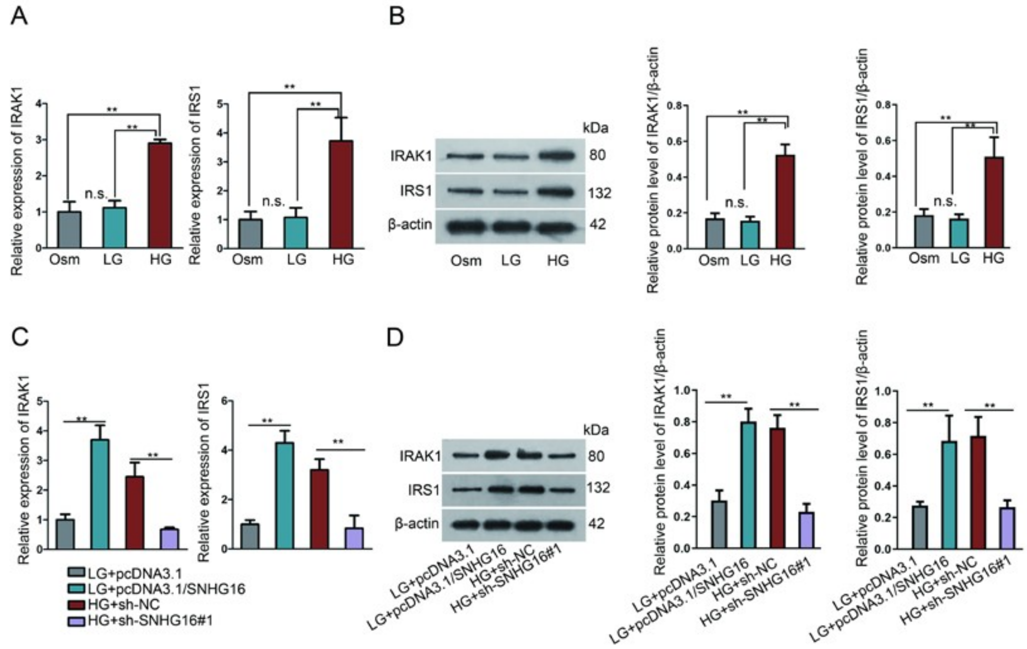

Figure S4 The effect of SNHG16 on NF-κB pathway activation through IRAK1. Experiments were conducted in cells treated with LG (5 mmol/L) for 48 hours. (A) Western blot analysis of IRAK1, p-IκBα and IκBα illustrated the change of IκBαphosphorylation level in response to IRAK1 knockdown or BAY 11-7082 treatment. N=3 in each group. (B) Western blot analysis of nuclear and cytoplasmic p65 illustrated the change of p65 nuclear translocation level in response to IRAK1 knockdown or BAY 11-7082 treatment. N=3 in each group. All data were acquired from three independent experiments and presented as the mean ± SD. $^{**}p < 0.01$, n.s. indicated difference was not statistically significant.

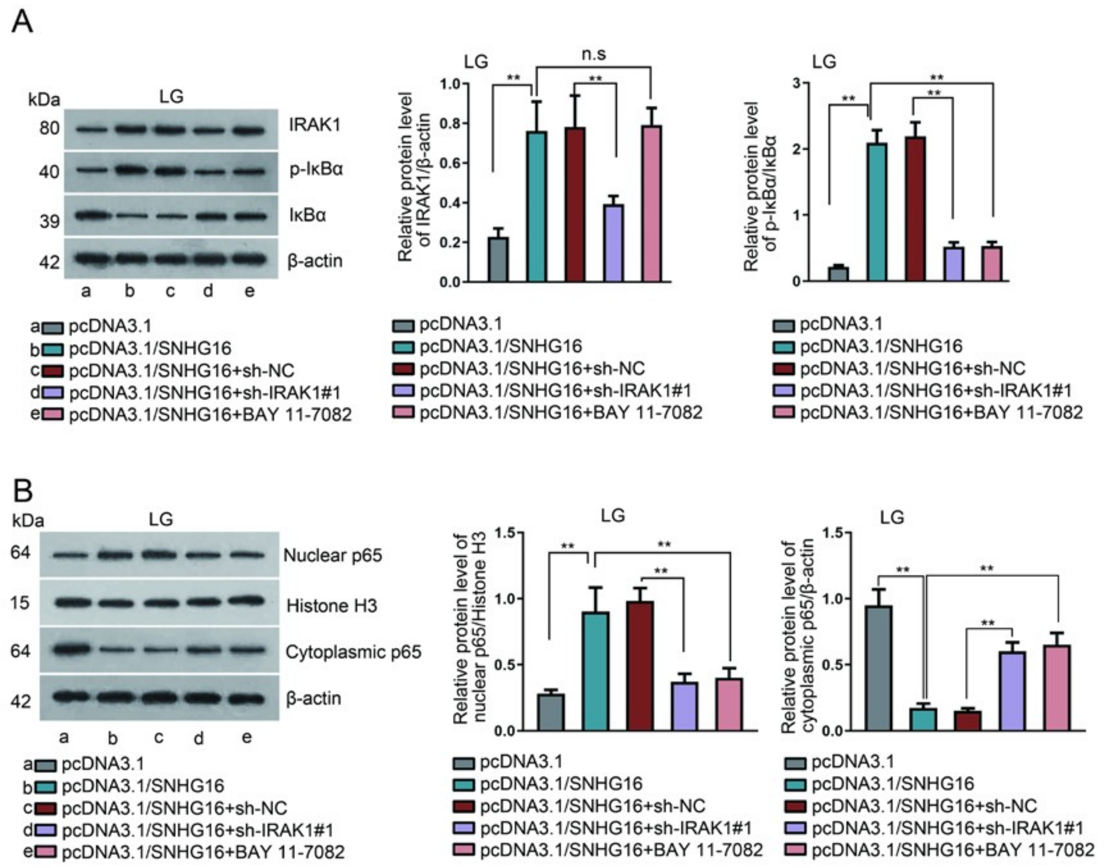

Figure S5 SNHG16 promotes hRMEC dysfunction via NF-κB pathway activation through IRAK1. Experiments were conducted in cells treated with LG (5 mmol/L) for 48 hours. (A and B) CCK-8 assay and EdU assay showed the effects of IRAK1 shRNAs and JSH-23 on cell proliferation in SNHG16-overexpressed hRMECs. N=3 in each group. (C and D) Wound healing assay and Transwell assay showed the effects of sh-IRAK1#1 transfection and JSH-23 addition on cell migration in hRMECs after SNHG16 overexpression. N=3 in each group. (E and F) qRT-PCR and ELISA showed the effects of sh-IRAK1#1 transfection and JSH-23 addition on VEGF level in hRMECs after SNHG16 overexpression. N=3 in each group. (G) Tube formation assay showed the effects of sh-IRAK1#1 transfection and JSH-23 addition on angiogenesis in SNHG16-overexpressed hRMECs. N=3 in each group. (H) ELISA-based NF-κB activity assay showed the change of NF-κB activity in response to IRAK1 knockdown or JSH-23 treatment. N=3 in each group. All data were acquired from three independent experiments and presented as the mean ± SD. $^{*}p < 0.05$, $^{**}p < 0.01$.

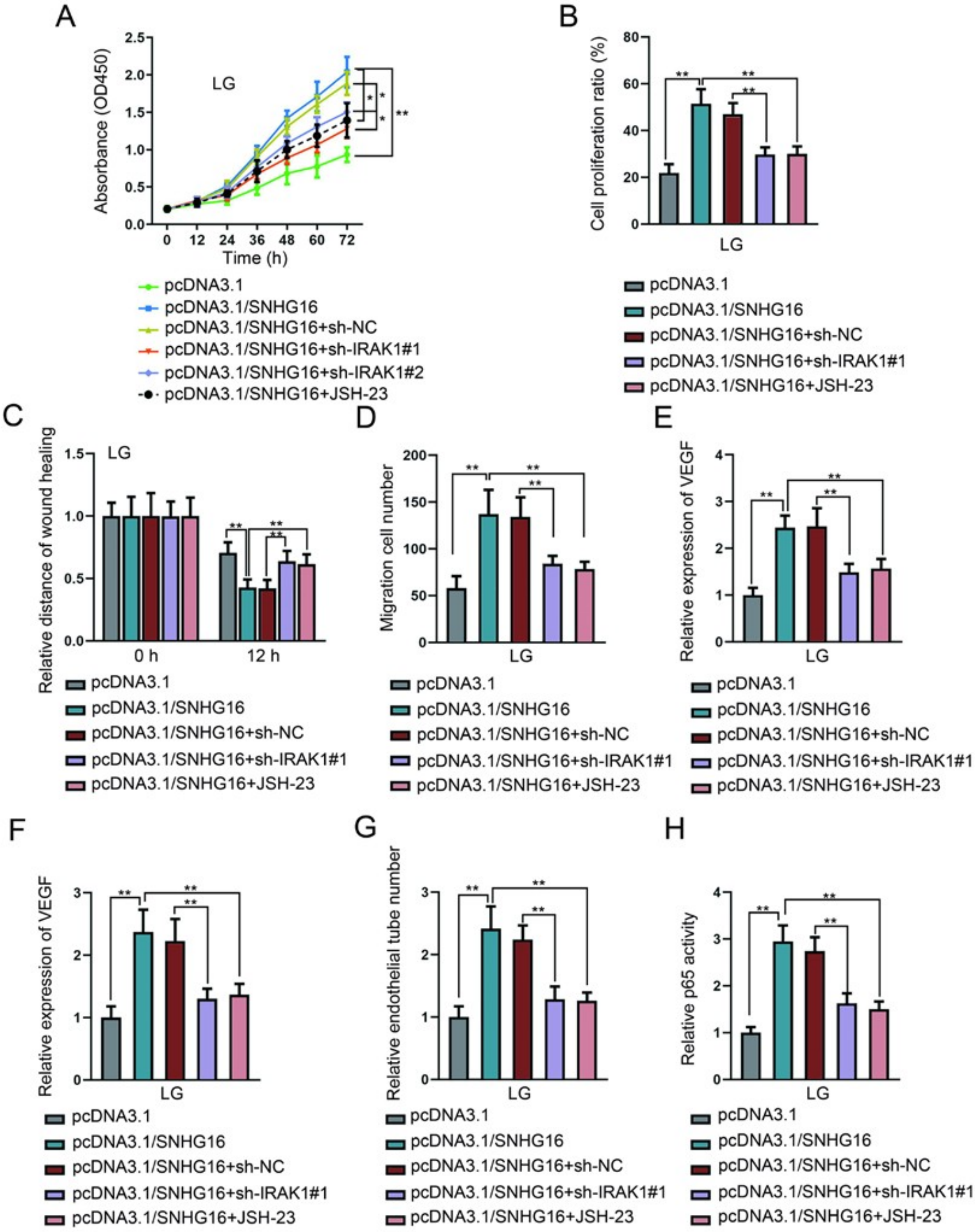
A
LG
Absorbance (OD450)
Time (h)
pcDNA3.1
pcDNA3.1/SNHG16
pcDNA3.1/SNHG16+sh-NC
pcDNA3.1/SNHG16+sh-IRAK1#1
pcDNA3.1/SNHG16+sh-IRAK1#2
pcDNA3.1/SNHG16+JSH-23
B
Cell proliferation ratio (%)
LG
pcDNA3.1
pcDNA3.1/SNHG16
pcDNA3.1/SNHG16+sh-NC
pcDNA3.1/SNHG16+sh-IRAK1#1
pcDNA3.1/SNHG16+JSH-23
C
LG
Relative distance of wound healing
0 h
12 h
pcDNA3.1
pcDNA3.1/SNHG16
pcDNA3.1/SNHG16+sh-NC
pcDNA3.1/SNHG16+sh-IRAK1#1
pcDNA3.1/SNHG16+JSH-23
D
Migration cell number
LG
pcDNA3.1
pcDNA3.1/SNHG16
pcDNA3.1/SNHG16+sh-NC
pcDNA3.1/SNHG16+sh-IRAK1#1
pcDNA3.1/SNHG16+JSH-23
E
Relative expression of VEGF
LG
pcDNA3.1
pcDNA3.1/SNHG16
pcDNA3.1/SNHG16+sh-NC
pcDNA3.1/SNHG16+sh-IRAK1#1
pcDNA3.1/SNHG16+JSH-23
F
Relative expression of VEGF
LG
pcDNA3.1
pcDNA3.1/SNHG16
pcDNA3.1/SNHG16+sh-NC
pcDNA3.1/SNHG16+sh-IRAK1#1
pcDNA3.1/SNHG16+JSH-23
G
Relative endothelial tube number
LG
pcDNA3.1
pcDNA3.1/SNHG16
pcDNA3.1/SNHG16+sh-NC
pcDNA3.1/SNHG16+sh-IRAK1#1
pcDNA3.1/SNHG16+JSH-23
H
Relative p65 activity
LG
pcDNA3.1
pcDNA3.1/SNHG16
pcDNA3.1/SNHG16+sh-NC
pcDNA3.1/SNHG16+sh-IRAK1#1
pcDNA3.1/SNHG16+JSH-23

Figure S6 The effect of SNHG16 on NF-κB pathway activation. Experiments were conducted in cells treated with LG (5 mmol/L) for 48 hours. (A) Western blot analysis of IRAK1, p-IκBα and IκBα illustrated the change of IκBα phosphorylation level in response to IRAK1 knockdown or JSH-23 treatment. N=3 in each group. (B) Western blot analysis of nuclear and cytoplasmic p65 illustrated the change of p65 nuclear translocation level in response to IRAK1 knockdown or JSH-23 treatment. N=3 in each group. All data were acquired from three independent experiments and presented as the mean ± SD. **p < 0.01, n.s. indicated difference was not statistically significant.

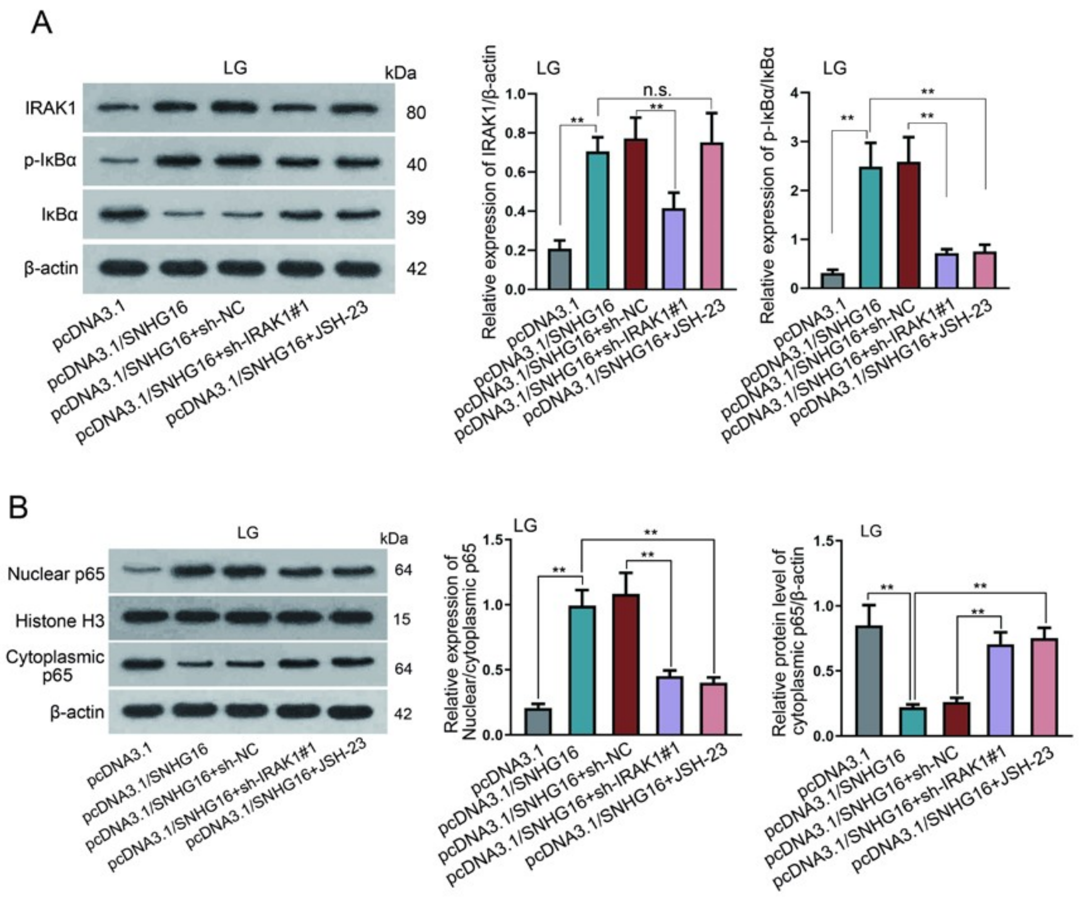

Figure S7 SNHG16 regulates PI3K/AKT pathway in LG-induced hRMECs. Experiments were conducted in cells treated with LG (5 mmol/L) for 48 hours. (A) Western blot analysis of IRS1, PI3K, p-AKT and AKT illustrated the change of PI3K/AKT pathway activity in response to IRS1 knockdown or LY294002 treatment. N=3 in each group. All data were acquired from three independent experiments and presented as the mean ± SD. **p < 0.01, n.s. indicated data was not statistically significant.

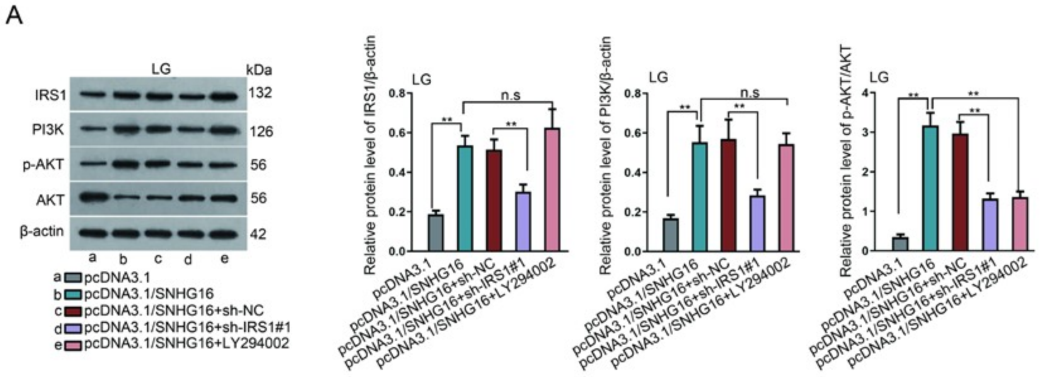

Figure S8 SNHG16 promotes hRMEC dysfunction via PI3K/AKT pathway activation through IRS1. Experiments were conducted in cells treated with LG (5 mmol/L) for 48 hours. (A and B) CCK-8 assay and EdU assay showed the effects of IRS1 shRNAs and ZSTK474 on cell proliferation in SNHG16-overexpressed hRMECs. N=3 in each group. (C and D) Wound healing assay and Transwell assay showed the effects of sh-IRS1#1 transfection and ZSTK474 addition on cell migration in hRMECs after SNHG16 overexpression. N=3 in each group. (E and F) qRT-PCR and ELISA showed the effects of sh-IRS1#1 transfection and ZSTK474 addition on VEGF level in hRMECs after SNHG16 overexpression. N=3 in each group. (H) Tube formation assay showed the effects of sh-IRS1#1 transfection and ZSTK474 addition on angiogenesis in SNHG16-overexpressed hRMECs. N=3 in each group. All data were acquired from three independent experiments and presented as the mean ± SD.

*$p < 0.05$, **$p < 0.01$.

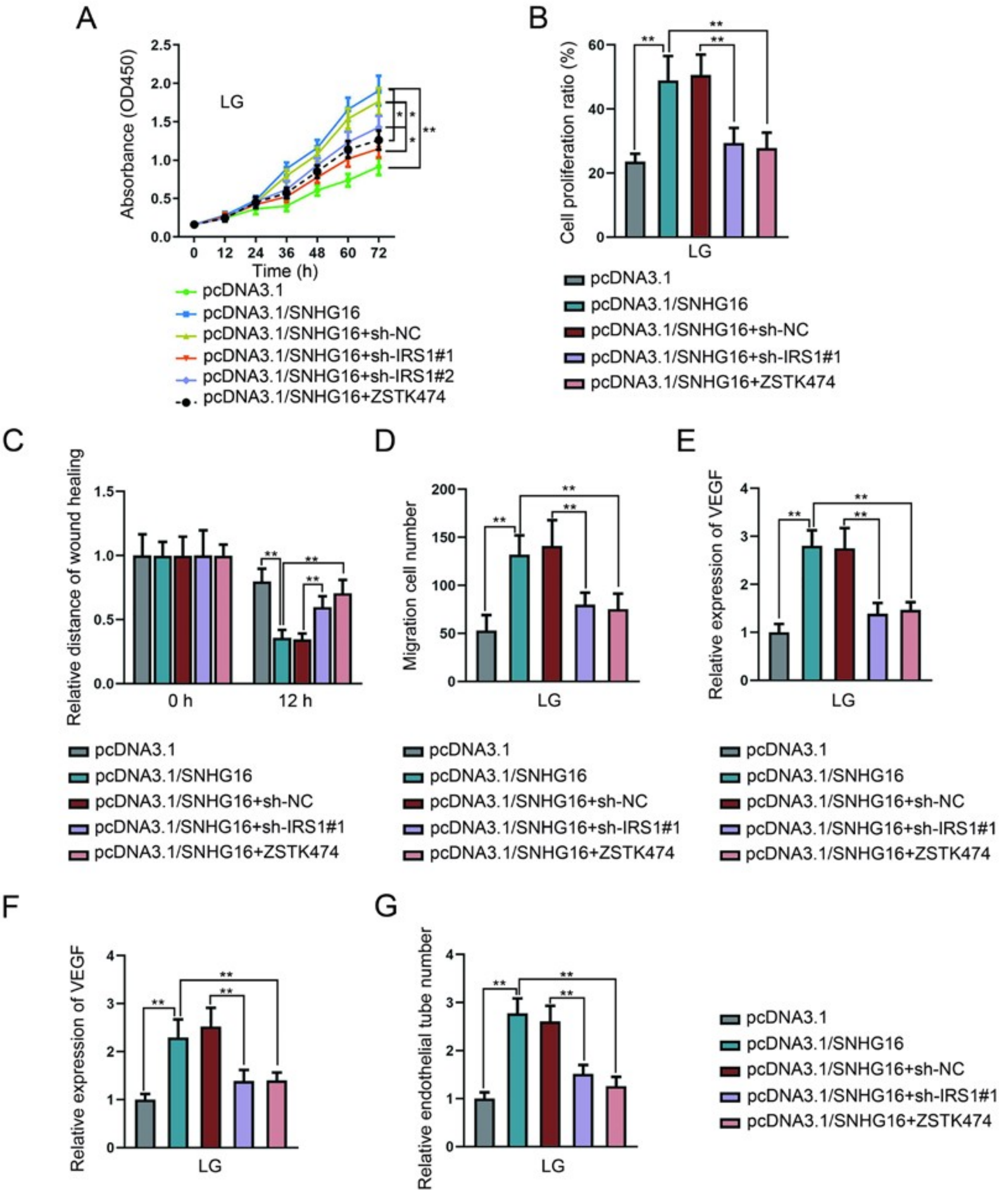

A
B
C
D
E
F
G
LG
Absorbance (OD450)
Time (h)
Cell proliferation ratio (%)
Relative distance of wound healing
0 h
12 h
Migration cell number
Relative expression of VEGF
Relative endothelial tube number
pcDNA3.1
pcDNA3.1/SNHG16
pcDNA3.1/SNHG16+sh-NC
pcDNA3.1/SNHG16+sh-IRS1#1
pcDNA3.1/SNHG16+sh-IRS1#2
pcDNA3.1/SNHG16+ZSTK474

Figure S9 SNHG16 regulates PI3K/AKT pathway in LG-induced hRMECs. Experiments were conducted in cells treated with LG (5 mmol/L) for 48 hours. (A) Western blot analysis of IRS1, PI3K, p-AKT and AKT illustrated the change of PI3K/AKT pathway activity in response to IRS1 knockdown or ZSK474 treatment. N=3 in each group. All data were acquired from three independent experiments and presented as the mean ± SD. **p < 0.01, n.s. indicated difference was not statistically significant.

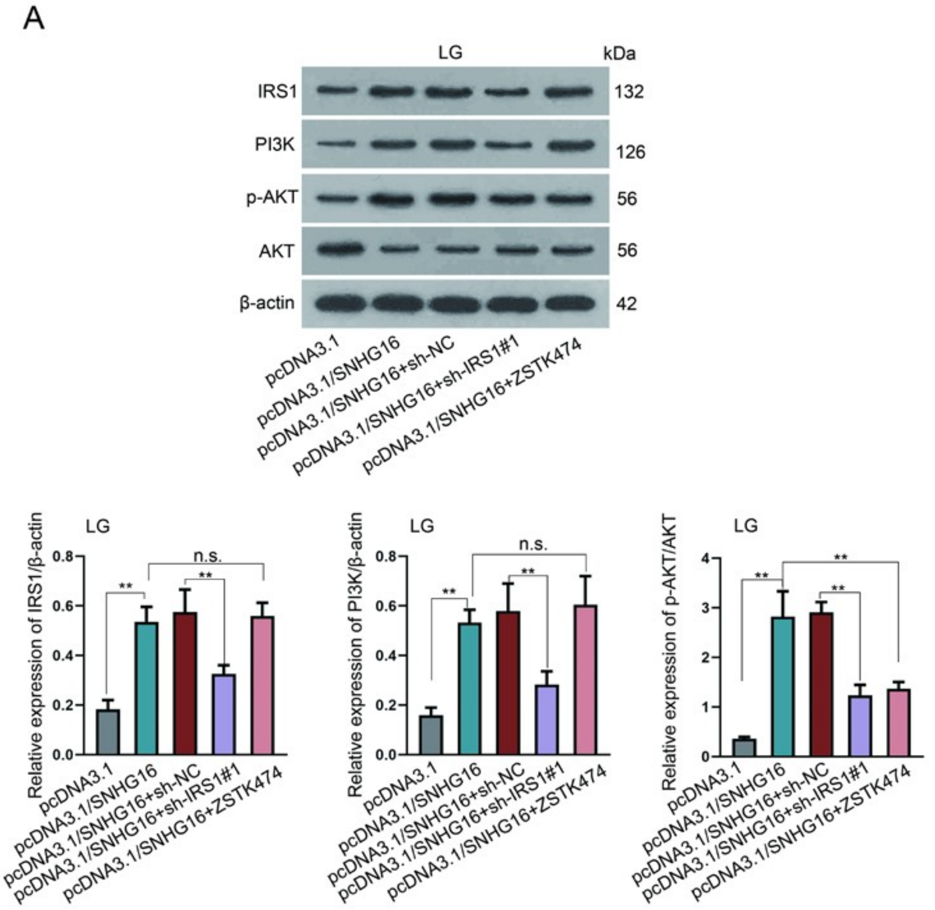

Table S1 qRT-PCR primers.

Gene ID	Primer	Sequence (5′-3′)
SNHG16	Forward	GCTGTGGCCTTGAAAACAGTC
	Reverse	GGGAGCTAACTCACATTAAAGACA
VEGF	Forward	AAGGAGGAGGGCAGAATCAT
	Reverse	ATCTGCATGGTGATGTTGGA
miR-146a-5p	RT	CTCAACTGGTGTCGTGGAGTCGGCAATTCAGTTGAGCAACCCA
	Forward	GCCGAGTGAGAACTGAATTCCA
miR-7-5p	RT	GTCGTATCCAGTGCAGGGTCCGAGGTATTCGCACTGGATACGACAAC
	Forward	CGCTGGAAGACTAGTGATTTT
Universal Primer	Reverse	CTCAACTGGTGTCGTGGA
IRAK1	Forward	TCAGAACGGCTTCTACTGCCTG
	Reverse	TACCCAGAAGGATGTCCAGTCG
IRS1	Forward	CTTCTCAGACGTGCGCAAGG
	Reverse	GTTGATGTTGAAACAGCTCTC
CARD10	Forward	CTTCGTCCTCATCCTCTGACAG
	Reverse	AGACACCCGAATAGCCAGAGAC
TRAF6	Forward	CAATGCCAGCGTCCCTTCCAAA
	Reverse	CCAAAGGACAGTTCTGGTCATGG
GAPDH	Forward	GGAGCGAGATCCCTCCAAAAT
	Reverse	GGCTGTTGTCATACTTCTCATGG
U6	Forward	CTCGCTTCGGCAGCACA
	Reverse	AACGCTTCACGAATTTGCGT

Table S2 shRNA sequences and FISH probe sequence.

sh-NC:
CCGGTGAATCTCAACTACATGTTTGCTCGAGCAAACATGTAGTTGAGATTCATTTTTG

sh-SNHG16#1:
CCGGACATTTTCTCAACGTTTGGAACTCGAGTTCCAAACGTTGAGAAAATGTTTTTG

sh-SNHG16#2:
CCGGATCTGTAAAGCTAAATATGAACTCGAGTTCATATTTAGCTTTACAGATTTTTG

sh-SNHG16#3:
CCGGACAAAAGATACCATTTCAGACCTCGAGGTCTGAAATGGTATCTTTT

sh-NC:
CCGGGTGATGGTGATGAGTAGTCTACTCGAGTAGACTACTCATCACCATCACTTTTTG

sh-IRS1#1:
CCGGGGGTTTGGAGAATGGTCTTAACTCGAGTTAAGACCATTCTCCAAACCCTTTTTG

sh-IRS1#2:
GTACCGGACTCATTGCCAAGATCCTTTACTCGAGTAAAGGATCTTGGCAATGAGTTTTTTG

sh-IRS1#3:
CCGGGCCGCTCAAGTGAGGATTTAACTCGAGTTAAATCCTCACTTGAGCGGCTTTTTG

sh-NC:
CCGGACGTGATAGAGTACTGATCTACTCGAGTAGATCAGTACTCTATCACGTTTTTTG

sh-IRAK1#1:
CCGGTGGTGAAACAGAGCTTCTTAACTCGAGTTAAGAAGCTCTGTTTCACCATTTTTG

sh-IRAK1#2:
CCGGCAATTGCTGCCCAGATCTATACTCGAGTATAGATCTGGGCAGCAATTGTTTTTG

sh-IRAK1#3:
CCGGTACCGAGCAGTCATGAGAAATCTCGAGATTTCTCATGACTGCTCGGTATTTTTG

Sequence for SNHG16-probe used in FISH assay

ugacgguaguuucccaaguuuauuguaagugguuuuaaguuaagucucauccaaacaaguuaucacacagcacuuaaccaagcccugggauuuacugucuugaugacuacacggcuuugcacagucugagaugcuucagugugcaaggcagcagcuggggggggaggagggggggucuucacagggacagcuggcaagagacuuccugaggcacaucaguuacguuggucaauuuagggcacggucugguucugcagcuuugaaagguggauucuuucuauuagcacacuuuacaagagggauuguaaaggauuaacucagucaccagaaacgaaacaccacuucagaaauucagagaccucugaucaacagaacagacauuugggcuuuaacugcuaaagcagcuaccuacuuggggaaaccauggcauucugcugcc

uggacagcaggaauuaagagagauuucagaguuacuggcacgaggacaaagccucucagcucgcuucaccuug
gcaaccuuaaacagucccuccuuggucucuaugauagcaguauguuccuuuggauggaauacuugguuugga
aauaauguagucaaauguaggauauguaaguacucccucaacagucacaauccauccuaguaucuuaaauagug
auuuuuuuuuuuaauuuacaaaagagguuuauuggacuuacaguucuacauggcugggaaggccucacaau
cauggcggaaggugaaagccacaucucacauggcagcagauaagagaaaaaaagguagugaucuuuuaguaaa
gaaaccugagacugguagugggcuuggagccagaggaucgcuuaaguccgggaguucgagaccagccagggu
aacaaauugagacccccccccaacuuuaaaauuaaaaaacgaaagaaaaaauagcugggugugguggcucacac
cuguaauccuagcacuuugggaggccaaggcggguggauugccugagcucaggaguuugagaccaaccuggg
caacaugguugaaacccugucucuacuaaaacaaaaaaaaucagccaggugugguagcgugcaccuguaguccc
aguuacuugggaggcugaggcacaagaauugcuugaaccugggagguagagguugcagagaucaugccacug
cacucccagccugggugacagagcaagacugucucaaaaacaacaacaacaacaacaaaacaaaacaaaaaaa
accucucaaaaaaaugaaaaaaaauuuaaauuuaaauuaaaaaaaaaaaugcugggucugauggcucacaccug
ucaucucagcacuuugggaagcugaggcaggcagaucacaaggucaggagauagagaccauccuggccaacau
ggugaaacccagucucuacuaaagaagguacgcgccuguagucccagcacucaggaggcugagccaggagaau
ugcuugaacccgggagguggagguugcagugagccaagauuguaccacuucacuccagccuggcgacagagug
gaacuccgucucaaaaaauaaaauaaaauaaaauaaaauaaaauaaaauaaaauaaaauaaagcaaaaauauaaa
auguuaaaaaaaaaacaaaaaaagggaaaaaggaagcugauugccuuggugagucaacacuggguauuuucug
accacuauuugaaacaaaaaaggaaaccacugauauucuaugcaaagaucuguuccuggaaggcacucugcgga
gacaccaggagaacuuuuaucaauccuucauugauuugaaguaaaagugcuaaagcaaugguuguggguggca
acccauuagcagaucacaaaaucacuguagugggguaacuaaacaagaggaaacacaagacggcauccuguguaa
cugggguuaagcauuacucucugaaacucauggcaucaguuuccucuuaggcucuuccaacagaguauaauca
uguucauuucaguuuacaauccuugcagucccaucgauuugugagauuauacccaggucauccacaguggagg
ucugaaaauguucuuaguuguacucuaauuucacuaacugccaaaagguuuuccagaauaaucucaguugcuu
cauuccuuuaaagaugaagcccaaagaacgcauggcgauuacuuuagaggaacaauuagcagcagaggcaggg
cugugcugaucccaucuggcaucgcugggagcuaacauuaaagacauggcacuuuggguccggguccaggucc
ugguucagagcagcugccacaccguggcuacuagaggauccuuuuccggcuuuggaaacugaggcugacugca
ccauuuacagaaauuucaucaugaagauccuuacacuuaacaccuuaaggugaugaguuuucacaugacuguu
uucaaggccacagccaccaccuuuaaucuaaaagagaaggauaacaggcuucauucuaacagaaaccaccuuaa
gguuuauucuccagauuuguauuaaacagcugagccucagcaagucagaccuccucacaaaagauaccauuuc
agacagggauaacuuuaaaucauccucagcucagccaauaacaaagauuccuaaauccuagcgauccaaaauuc
auuugccaugucucuuucaucccugcaugcccuaacgaggauccacgcugguuacaaugacaccuaguggccc
caaaucacacgggcaaagacgcuacucauuuuuaucauuaaaauaauuagcauucugaauuuugaguaauuuc
uuaccauagcuggcuuguaugucaacacguauucucauacuugaacuucagaauaaagcaggaggugaaugua
ugugauggcaccaacugugcuuucucauuucuagugaccacucaggcccugccugcacccacucuuacucaug
uuaagggcuccaucuauuacucugauccuucaaccuggagcucugcuuuucaccgucucugcagaugccaaac
aaagaaaggccucugggggaggaaaaaggcucguuguuaaaacuacggcuuuccagaaguuuacaauccuau
ugacuccuaucuguaaagcuaaauaugaacuaccgauuggggccguccucaagacacuccaagcggaaucacg
ucuucacacguuaacacuccugaugcugaauuaagaaccaccaucgucagagucugcccgugcgcgggcaccg
aagaccccugggcaugacgcucagcacacgcgcggcggaggcgcacggggcugcccagguuguuaggcgcucu
cccccgcagaagccggcccuaugacgccgaggacgggcuugagcucucucccuucacauaguaacauuuucuc
aacguuuggaaagcauuuucucauccaguacuuuuaacccuccuaaccacccuaugagggagaaaccgagauu
aucgucaccacuucgucucugaggaagggaagccaaguauguuagugucgugucaaggaagaccccguuuucc
cacccaggcagccuuaccaucaucacuaaaggccugagacugcucgccgugcucaacaccgacuggaguggcca
ugucuuccagccacgcggccgac
