# Supplementary material for: Upregulation of long non-coding RNA SNHG16 promotes diabetes-related RMEC dysfunction via activating NF-κB and PI3K/AKT pathways
Source: Mol Ther Nucleic Acids. 2021 Feb 4;24:512–27. doi: 10.1016/j.omtn.2021.01.035 (PMC8056184; doi:10.1016/j.omtn.2021.01.035)
Supplement: Document S2. Article plus supplemental information [file mmc2.pdf]

Molecular Therapy
Nucleic Acids
Original Article

Upregulation of long non-coding RNA SNHG16 promotes diabetes-related RMEC dysfunction via activating NF-κB and PI3K/AKT pathways

Fei Cai,[1,3] Huanzong Jiang,[1,3] Yan Li,[2] Qin Li,[1] and Chao Yang[1]

[1]Department of Vascular Surgery, Union Hospital, Tongji Medical College, Huazhong University of Science and Technology, Wuhan 430022, China; [2]Operation Room, Tongji Hospital, Tongji Medical College, Huazhong University of Science and Technology, Wuhan 430022, China

Diabetic retinopathy (DR) is a severe diabetes-induced eye disease, in which its pathological phenomena basically include abnormal proliferation, migration, and angiogenesis of microvascular endothelial cells in the retina. Long non-coding RNAs (lncRNAs) have been proven to be important regulators in various biological processes, but their participation in DR remains largely undiscovered. In the present study, we aimed to unveil the role of lncRNA small nucleolar RNA host gene 16 (SNHG16) in regulating the functions of human retinal microvascular endothelial cells (hRMECs) under a high-glucose (HG) condition. We found that SNHG16 expression was significantly upregulated in hRMECs treated with HG. Functionally, SNHG16 could facilitate hRMEC proliferation, migration, and angiogenesis. Moreover, SNHG16 was associated with nuclear factor κB (NF-κB) and phosphatidylinositol 3-kinase (PI3K)/AKT pathways. Mechanistically, SNHG16 could promote hRMEC dysfunction by sequestering microRNA (miR)-146a-5p and miR-7-5p to act as a competing endogenous RNA (ceRNA) with interleukin-1 receptor-associated kinase 1 (IRAK1) and insulin receptor substrate 1 (IRS1). In conclusion, our results illustrated the potential role of SNHG16 in facilitating hRMEC dysfunction under HG treatment, providing a novel approach for DR therapy.

INTRODUCTION

Diabetic retinopathy (DR) is a typical microvascular complication of diabetes mellitus. This serious oculopathy is prominent as the major cause of vision loss and blindness in diabetes patients, thereby impairing their quality of life.[1,2] In recent years, DR has become a worldwide public health issue with the sustained rise of diabetes occurrence, highlighting the significance of this severe disease.[3] In spite of the adoption of many techniques for DR management, such as laser surgery, intraocular drug injection, and vitrectomy, the lesions of DR can hardly be thoroughly cured through these conventional treatments.[4] Therefore, the underlying molecular mechanisms of DR pathogenesis need to be further investigated in order to exploit more novel and effective therapeutic strategies.

Retinal endothelial cells lining the microvascular system are critical in maintaining normal functions of retina. Besides, dysfunction of retinal endothelial cells plays an important role in the progression of many vasculopathies, including DR.[5] Specifically, the prevalence of DR is closely associated with diabetes duration, hypertension, and hyperglycemia. These risk factors can lead to pathological endothelial cell dysfunction, such as excessive proliferation, migration, and angiogenesis of endothelial cells. These abnormal behaviors can subsequently result in pathological retinal neovascularization during the proliferative stage of DR, which can gradually develop into blurred vision, severe visual impairment, and eventually result in blindness.[6–8] Therefore, our research was aimed at exploring the mechanism in regulating the behaviors of human retinal microvascular endothelial cells (hRMECs) cultured under high-glucose (HG) stimulation, imitating DR circumstances.

Long non-coding RNAs (lncRNAs), commonly defined to be long RNA transcripts (>200 nucleotides in length) with poor or no protein-coding capacity,[9,10] can involve in many physiological and pathological processes.[11] Although the participation of lncRNAs in DR remains largely undiscovered, increasing studies have been elucidating significant regulatory functions of various lncRNAs in vascular diseases and diabetes with different molecular mechanisms implicated.[12,13] For instance, lncRNA myocardial infarction-associated transcript (MIAT) regulates endothelial cell function in DR through a feedback loop with vascular endothelial growth factor (VEGF) and microRNA (miR)-150-5p.[14] MEG3 knockdown aggravates diabetes-related retinal vessel dysfunction via activating the phosphatidylinositol 3-kinase (PI3K)/AKT signaling pathway.[15] H19 prevents transforming growth factor (TGF)-β1-mediated endothelial-mesenchymal transition of retinal endothelial cells through the mitogen-activated

Received 19 May 2020; accepted 25 January 2021;
https://doi.org/10.1016/j.omtn.2021.01.035.

[3]These authors contributed equally

Correspondence: Qin Li, Department of Vascular Surgery, Union Hospital, Tongji Medical College, Huazhong University of Science and Technology, No. 1277 Jiefang Avenue, Wuhan 430022, China.
E-mail: li_qin@hust.edu.cn
Correspondence: Chao Yang, Department of Vascular Surgery, Union Hospital, Tongji Medical College, Huazhong University of Science and Technology, No.1277 Jiefang Avenue, Wuhan, 430022, China.
E-mail: ychao@hust.edu.cn

 © 2021 The Author(s).
This is an open access article under the CC BY license (http://creativecommons.org/licenses/by/4.0/).

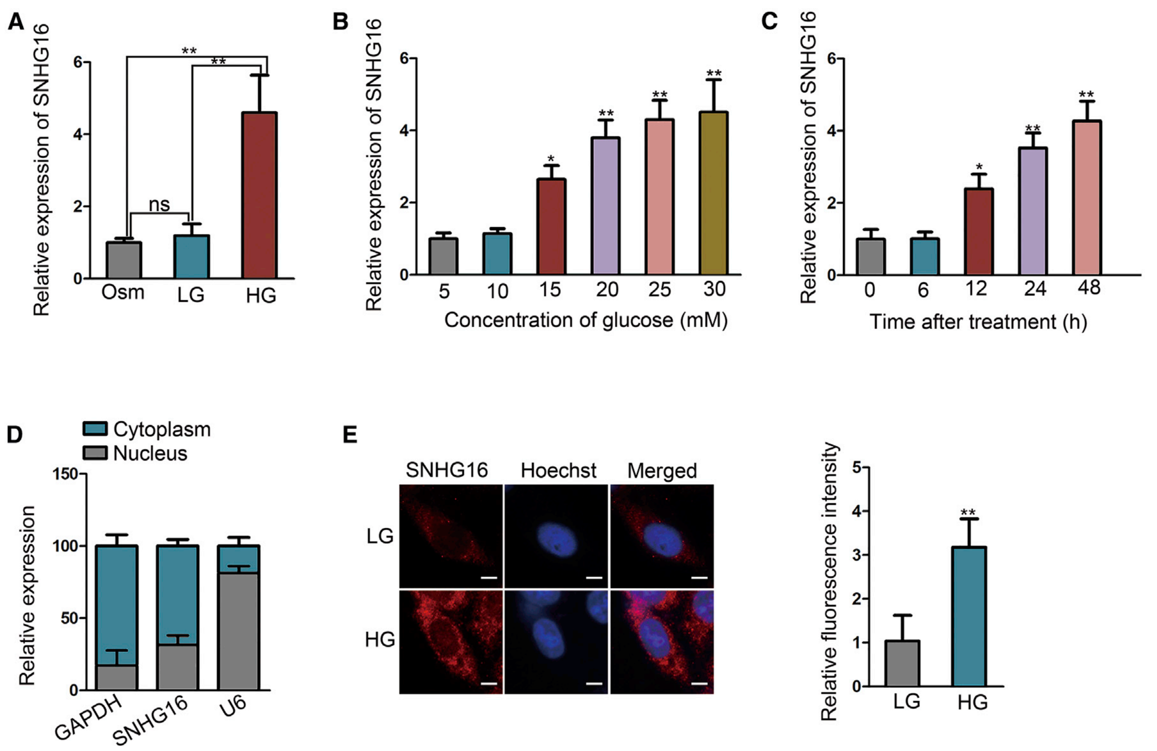

Figure 1. SNHG16 expression is upregulated in hRMECs under high-glucose (HG) condition

(A) SNHG16 expression in hRMECs cultured under different conditions was detected using quantitative real-time PCR, showing the upregulation of SNHG16 in hRMECs stimulated with HG (25 mM D-glucose) in comparison with low-glucose (LG; 5 mM D-glucose) or osmotic control (Osm; 25 mM L-glucose) groups. n = 3 in each group. (B and C) Multiple trials of quantitative real-time PCR showed that the SNHG16 level in hRMECs was increased in a glucose dose-dependent pattern (treated for 48 h) and in a culturing time-dependent pattern (25 mM). n = 3 in each group. (D) SNHG16 expression in the cytoplasm and nucleus of hRMEC using quantitative real-time PCR following hRMEC subcellular fractionation. GAPDH and U6 served as cytoplasmic and nuclear markers, respectively. n = 3 in each group. (E) SNHG16 subcellular distribution in hRMECs under LG or HG condition for 48 h was visualized using FISH (scale bars, 20 μm), in which data quantification was recorded as mean fluorescence intensity of SNHG16 probes accordingly. n = 3 in each group. All data were acquired from three independent experiments and presented as the mean ± SD. *$p < 0.05$, **$p < 0.01$, ns, difference was not statistically significant.

protein kinase (MAPK)-extracellular signal-regulated kinase (ERK) 1/2 pathway during DR progression.[16] Since, to date, only a few types of lncRNAs have been thoroughly identified as key molecular targets for DR therapy, further studies are still required for uncovering the participation of specific lncRNAs in regulating DR progression.

In this study, we focused on lncRNA small nucleolar RNA (snoRNA) host gene 16 (SNHG16), which can facilitate proliferation, migration, and angiogenesis of hemangioma endothelial cells.[17] Based on this discovery, we explored the potentiality of SNHG16 in regulating these pathological behaviors in RMECs and discussed the underlying molecular mechanism by which SNHG16 could exert its function in hRMECs.

RESULTS

SNHG16 expression is upregulated in hRMECs under HG condition

At first, we evaluated cell viability and proliferation under HG condition for 12 h and 48 h. It was found that the viability and proliferation were strengthened at 48 h (Figures S1A and S1B). Thus, we chose 48 h for subsequent experiments. The morphology of hRMECs treated with low glucose (LG) or HG was observed under a microscope. Cells become spindle in the HG group compared with the LG group (Figure S1C). To explore the participation of SNHG16 in diabetes-induced retinal endothelial cell dysfunction, we examined the expression level of different isoforms of SNHG16 in hRMECs cultured under HG or LG condition for 48 h, imitating diabetic and normal circumstances, respectively. The result revealed that the expression of SNHG16-201 (named SNHG16 in subsequence) was upregulated in the HG group (Figure S2A). Importantly, SNHG16 expression presented no statistical significance between LG and osmotic control (Osm) groups (Figure 1A). Additionally, through a series of trials, we found that the HG-induced elevation of the SNHG16 level was associated with the increase of glucose concentration (Figure 1B) or culturing time (Figure 1C). Subsequently, we detected the subcellular distribution of SNHG16 in hRMECs by performing subcellular fractionation and fluorescence *in situ* hybridization (FISH) assays. The results showed that SNHG16 was located mostly in the cytoplasm

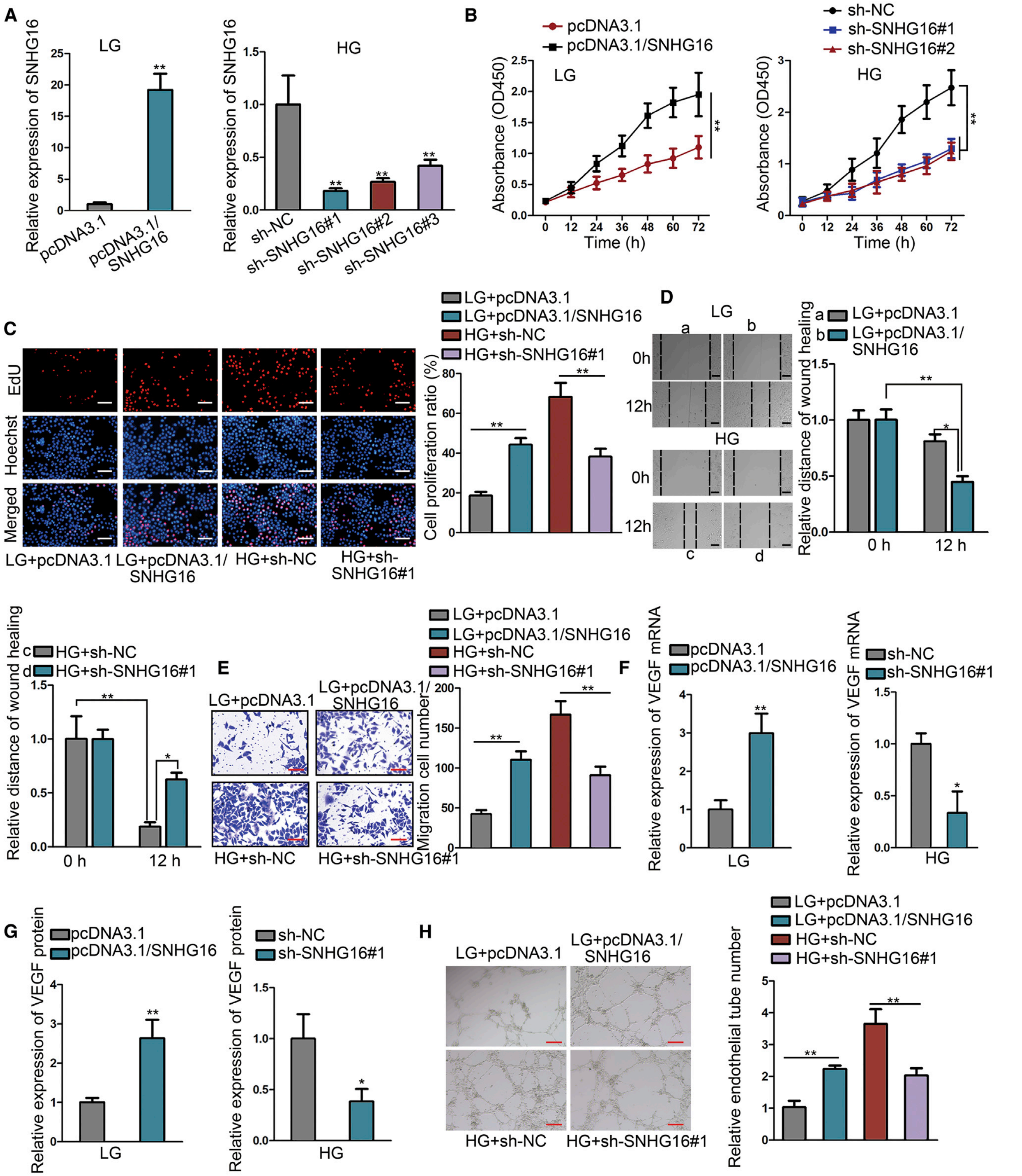

Figure 2. SNHG16 positively regulates proliferation, migration, and angiogenesis of hRMECs

Experiments were conducted in cells treated with LG (5 mM) or HG (25 mM) for 48 h. (A) Efficiency of SNHG16 overexpression in LG-treated hRMECs and of SNHG16 knockdown in HG-treated hRMECs was assessed using quantitative real-time PCR. Two shRNAs for SNHG16 with relatively higher knockdown capacity were selected.

(legend continued on next page)

of hRMECs. Furthermore, as illustrated by relative fluorescence intensity of FISH probes, the higher level of SNHG16 in HG-treated hRMECs could also been observed (Figures 1D and 1E). These results indicated that SNHG16 upregulation was associated with HG condition, suggesting the potentiality of SNHG16 in aggravating diabetes-related hRMEC dysfunction.

SNHG16 encodes three snoRNAs. Here, we also investigated whether SNHG16 could regulate these three snoRNAs and thus led to hRMEC dysfunction. As shown in Figure S2B, there were no significant differences of the expression of three snoRNAs between LG and HG groups. In addition, overexpression of SNHG16 in LG-induced hRMECs and knockdown of SNHG16 in HG-induced hRMECs had no significant effect on the expression of three snoRNAs (Figure S2C). Subsequently, we performed functional assays to demonstrate the role of three snoRNAs in modulating hRMEC functions. As a result, silencing of these three snoRNAs had no effects on the functions of hRMECs (Figures S2D–S2J). Therefore, we excluded the possibility that SNHG16 exerts functions through modulating its snoRNAs.

SNHG16 positively regulates proliferation, migration, and angiogenesis of hRMECs

To evaluate the impact of SNHG16 overexpression or knockdown on hRMEC functions, we performed gain-of-function and loss-of-function assays by transfecting pcDNA3.1/SNHG16 overexpression constructs into LG-treated hRMECs and lentiviral vectors with short hairpin RNAs (shRNAs) targeting SNHG16 into HG-treated hRMECs, respectively (Figure 2A). First, we performed Cell Counting Kit 8 (CCK-8) and 5-ethynyl-2′-deoxyuridine (EdU) assays to analyze the cell proliferation level. The result showed that hRMEC proliferation was significantly promoted by SNHG16 overexpression and inhibited by SNHG16 knockdown, as illustrated by the absorbance at 450 nm in the CCK-8 assay (Figure 2B). The same tendency was shown by measuring the ratio of EdU-positive cells (Figure 2C). Additionally, we uncovered that HG treatment induced the decrease of reactive oxygen species (ROS) level and suppressed cell apoptosis, whereas these tendencies were reversed by the silencing of SNHG16 (Figures S1D and S1E). Next, we detected hRMEC migration by conducting wound-healing and Transwell assays and found that SNHG16 overexpression significantly enhanced cell migration, whereas SNHG16 knockdown significantly suppressed cell migration (Figures 2D and 2E).

Furthermore, it is acknowledged that VEGF is an important angiogenic cytokine that can potently accelerate vascular endothelial cell proliferation, migration, as well as angiogenesis.[18] Therefore, we evaluated the influence of SNHG16 on mRNA and protein levels of VEGF. The result demonstrated that VEGF level was increased in hRMECs with SNHG16 overexpression and decreased by SNHG16 knockdown (Figures 2F and 2G). We also detected whether HG-induced SNHG16 affected the hypoxia-inducible factor (HIF)-1α/VEGF axis. The results indicated that the levels of HIF-1α and VEGF were increased in LG-treated hRMECs after SNHG16 overexpression. The levels of them were also enhanced by HG treatment but were reduced again by the knockdown of SNHG16 (Figure S1F). Based on the angiogenic role of VEGF, we analyzed the influence of SNHG16 on angiogenesis using a tube formation assay. The result showed the positive effect of SNHG16 overexpression and the inhibitory effect of SNHG16 knockdown on endothelial tube formation (Figure 2H). Collectively, the above results convincingly demonstrated that SNHG16 markedly promoted HG-induced hRMEC proliferation, migration, and angiogenesis.

SNHG16 is associated with nuclear factor κB (NF-κB) and PI3K/AKT pathways

To further explore the mechanism by which SNHG16 could regulate diabetes-related hRMEC dysfunction, we performed a series of transcription factor transactivation assays. The potential outcome of SNHG16 knockdown in HG-treated hRMECs was evaluated through analyzing the change of luciferase activity in each group of transcription factor, which represents a certain signaling pathway that may play a part in regulating hRMEC behaviors. Intriguingly, we observed that the transactivation levels of NF-κB and forkhead box O (FOXO), which, respectively, stand for NF-κB pathways and PI3K/AKT pathways, were dramatically decreased after SNHG16 knockdown, whereas the influences on other transcription factors were not significant (Figure 3A). This association was verified by the enhanced transactivation levels of NF-κB and FOXO in LG-treated hRMECs with SNHG16 overexpression (Figure 3B).

Next, we explored whether the levels of key molecules involved in NF-κB or PI3K/AKT pathways were affected by the SNHG16 level. In LG-treated hRMECs, SNHG16 overexpression enhanced the phosphorylation level of the NF-κB inhibitory protein IκBα (p-IκBα), thus facilitating its degradation. In HG-treated hRMECs, contrarily, p-IκBα was inhibited by SNHG16 knockdown (Figure 3C). Then, we assessed the level of nuclear or cytoplasmic p65, the pivotal transcriptional regulation factor of NF-κB, and found that p65 nuclear translocation was promoted by SNHG16 overexpression, whereas SNHG16 knockdown induced p65 cytoplasmic retention (Figure 3D). Subsequently, we

n = 3 in each group. (B) CCK-8 assay was performed to assess the influence of SNHG16 overexpression or knockdown on hRMEC proliferation. The shRNA for SNHG16 with the strongest effect was adopted for all following experiments. n = 3 in each group. (C) Cell proliferation ratio of each group was illustrated through calculating the percentage of EdU-positive cells using the EdU assay (scale bars, 200 μm). n = 3 in each group. (D) Wound-healing assay illustrated the effect of SNHG16 overexpression or knockdown on hRMEC migration through measuring the wound-healing distance (scale bars, 100 μm). n = 3 in each group. (E) Migration cell number of each group was counted using Transwell assay. n = 3 in each group. (F) quantitative real-time PCR analysis of VEGF mRNA level influenced by SNHG16 overexpression or knockdown. n = 3 in each group. (G) ELISA showed VEGF protein level influenced by SNHG16 overexpression or knockdown. n = 3 in each group. (H) Tube formation assay was performed to evaluate the influence of SNHG16 overexpression or knockdown on angiogenesis (scale bars, 200 μm). n = 3 in each group. All data were acquired from three independent experiments and presented as the mean ± SD. *p < 0.05, **p < 0.01.

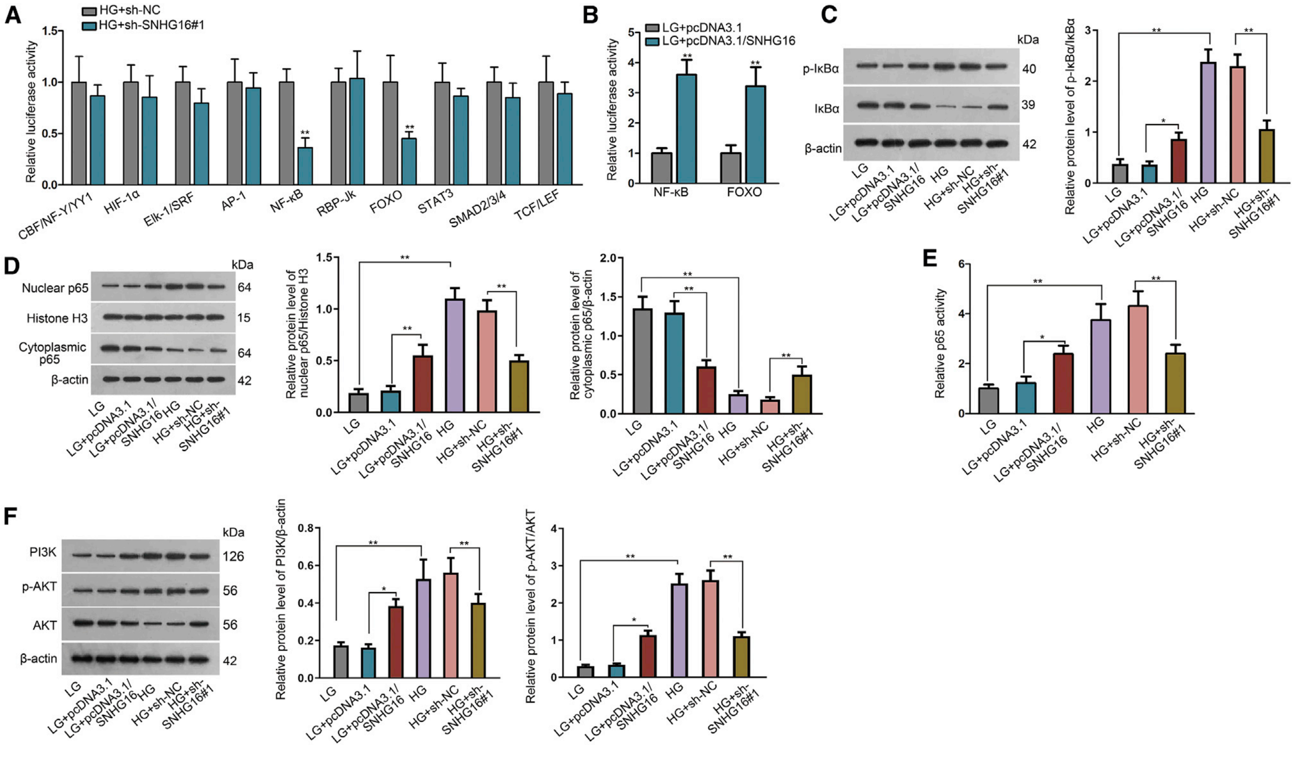

Figure 3. SNHG16 is associated with NF-κB and PI3K/AKT pathways

Experiments were conducted in cells treated with LG (5 mM) or HG (25 mM) for 48 h. (A) The downstream signaling pathway of SNHG16 was evaluated using Cignal Reporter Assay to detect the activities of a series of transcription factors in hRMECs with SNHG16 knockdown. The transcription factors listed above represent endoplasmic reticulum (ER) stress, hypoxia, MAPK/ERK, MAPK/c-Jun N-terminal kinase (JNK), NF-κB, Notch, PI3K/AKT, STAT3, TGF-β, and Wnt signaling pathways. n = 3 in each group. (B) Cignal Reporter Assay was performed to explore the signaling activity of NF-κB and PI3K/AKT in SNHG16-overexpressed hRMECs. n = 3 in each group. (C) Western blot analysis of p-IκBα and IκBα illustrated the effect of SNHG16 overexpression or knockdown on p-IκBα. n = 3 in each group. (D) Western blot analysis of nuclear and cytoplasmic p65 following subcellular fractionation illustrated the effect of SNHG16 overexpression or knockdown on p65 nuclear translocation. Histone H3: nuclear control; β-actin: cytoplasmic control. n = 3 in each group. (E) p65 activity in hRMECs with SNHG16 overexpression or knockdown was detected using ELISA-based NF-κB activity assay. n = 3 in each group. (F) Western blot analysis of PI3K, p-AKT, and AKT showed that SNHG16 positively regulated the PI3K/AKT pathway in hRMECs. n = 3 in each group. All data were acquired from three independent experiments and presented as the mean ± SD. *$p < 0.05$, **$p < 0.01$.

performed an enzyme-linked immunosorbent assay (ELISA) and found that the low p65 activity in LG-treated hRMECs was enhanced by SNHG16 overexpression, whereas the high p65 activity in HG-treated hRMECs was reduced by SNHG16 knockdown (Figure 3E). As for the PI3K/AKT pathway, after SNHG16 overexpression, the levels of PI3K expression and p-AKT were elevated, indicating the positive regulation of the PI3K/AKT pathway. In contrast, PI3K expression and p-AKT were decreased after SNHG16 knockdown (Figure 3F). These results suggested the activation of NF-κB and PI3K/AKT signaling pathways by SNHG16 in hRMECs.

SNHG16 directly interacts with miR-146a-5p and miR-7-5p

Accumulating evidence has elucidated that lncRNAs can act as competing endogenous RNAs (ceRNAs) to sponge specific microRNAs (miRNAs), thus protecting target transcripts from being degraded.[19,20] Since SNHG16 has been reported in many studies to serve as a ceRNA and take important part in various diseases,[21–23] and SNHG16 was located mainly in the cytoplasm of hRMECs, we assumed that the possible mechanism by which SNHG16 exerted its function in hRMECs was to sequester key miRNAs involved in NF-κB and PI3K/AKT pathways. Therefore, we resorted to the Encyclopedia of RNA Interactomes (ENCORI) online database (http://starbase.sysu.edu.cn/), a public bioinformatics tool[24] and searched out the putative target miRNAs for SNHG16. Among these, miR-146a-5p and miR-7-5p were selected as predicted candidate miRNAs, because they have been explicitly reported to, respectively, inhibit NF-κB and PI3K/AKT pathways in RMECs.[25–27] The predicted binding sites for miR-146a-5p or miR-7-5p on SNHG16 were also illustrated (Figure 4A).

Next, we found that in contrast with SNHG16, miR-146a-5p and miR-7-5p levels were markedly downregulated in HG-treated hRMECs (Figure 4B). Besides, SNHG16 overexpression led to reduced miR-146a-5p and miR-7-5p levels in hRMECs under LG condition, whereas SNHG16 knockdown resulted in elevated miR-146a-5p and miR-7-5p levels under HG condition, indicating that SNHG16 negatively regulated miR-146a-5p and miR-7-5p

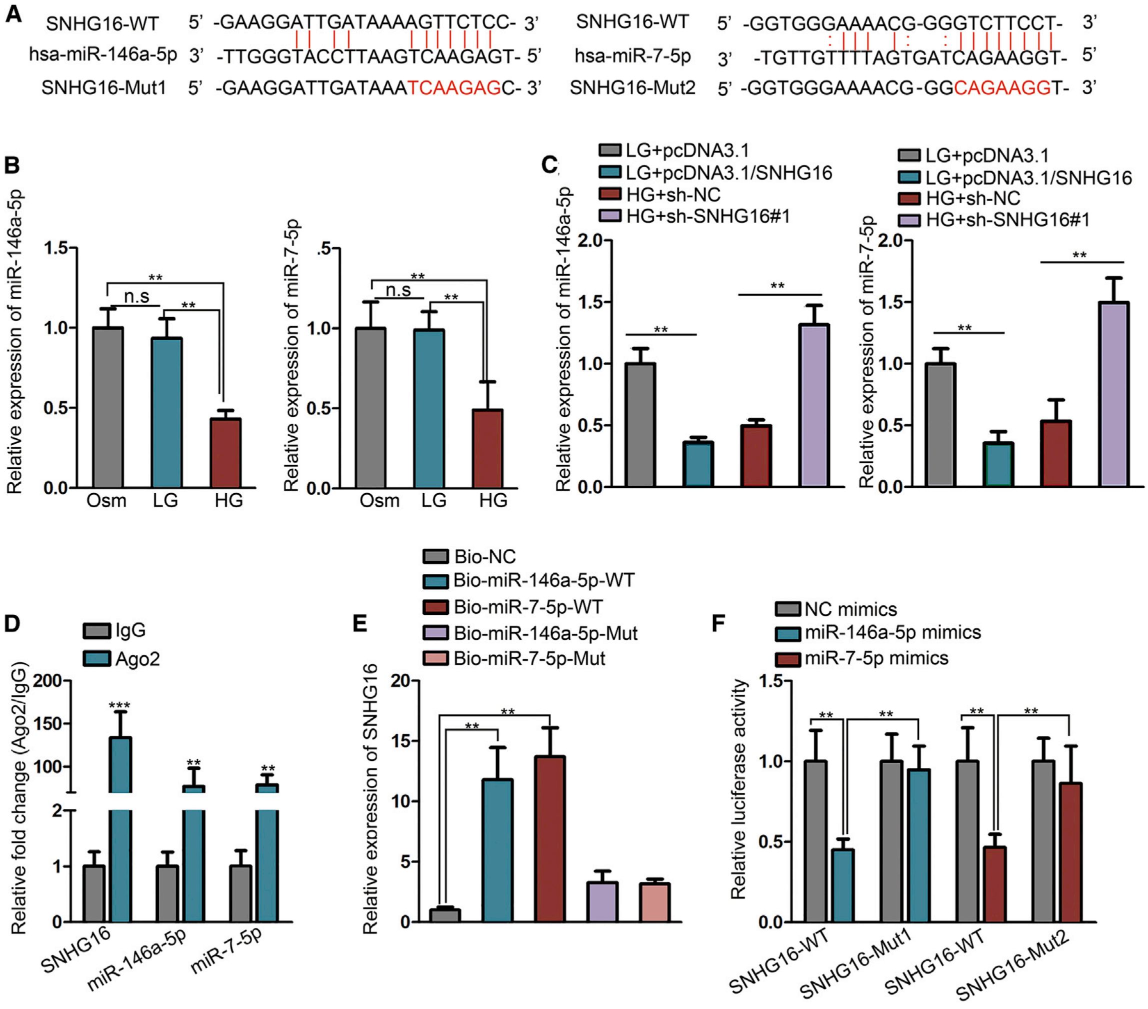

Figure 4. SNHG16 directly interacts with miR-146a-5p and miR-7-5p

Experiments were conducted in cells treated with LG (5 mM) or HG (25 mM) for 48 h. (A) ENCORI prediction showed the binding sites of miR-146a-5p and miR-7-5p in the sequence of SNHG16. The sequences highlighted in red stand for the mutant (Mut) binding sites of miR-146a-5p and miR-7-5p for luciferase reporter assay. (B) miR-146a-5p and miR-7-5p expression in hRMECs cultured with HG, LG, or Osm was detected using quantitative real-time PCR. n = 3 in each group. (C) Quantitative real-time PCR showed the levels of miR-146a-5p and miR-7-5p influenced by SNHG16 overexpression or knockdown. n = 3 in each group. (D) RIP assay with Ago2 antibody or IgG as a control, followed by quantitative real-time PCR, demonstrated the enrichment of SNHG16, miR-146a-5p, and miR-7-5p in RISCs. n = 3 in each group. (E) RNA pull-down assay, followed by quantitative real-time PCR, was performed to detect the level of SNHG16 interacting with miR-146a-5p or miR-7-5p. n = 3 in each group. (F) Luciferase reporter assay was performed in hRMECs co-transfected with SNHG16-WT or SNHG16-Mut1 luciferase reporters and miR-146a-5p mimics or NC mimics, as well as in hRMECs co-transfected with SNHG16-WT or SNHG16-Mut2 luciferase reporters and miR-7-5p mimics or NC mimics. n = 3 in each group. All data were acquired from three independent experiments and presented as the mean ± SD. **$p < 0.01$, ***$p < 0.001$.

(Figure 4C). Further, RNA immunoprecipitation (RIP) assay, using hRMEC lysates, was conducted, illustrating that SNHG16, miR-146a-5p, and miR-7-5p were enriched in Ago2 groups compared with immunoglobulin G (IgG) control groups, illustrating their recruitment to RNA-induced silencing complexes (RISCs) (Figure 4D). Besides, through RNA pull-down assay, the direct interaction between SNHG16 and miR-146a-5p or miR-7-5p was identified, since only by wild-type (WT) biotinylated miRNA probes could SNHG16 be significantly pulled down (Figure 4E). Moreover, in a luciferase reporter assay, luciferase activity of WT SNHG16 reporters was significantly reduced in response to miR-146a-5p or miR-7-5p, but luciferase activity of SNHG16 reporters with mutant (Mut) binding sites was not affected (Figure 4F). These results illustrated the direct interaction between SNHG16 and miR-146a-5p or miR-7-5p in hRMECs.

miR-146a-5p and miR-7-5p inhibitors reverse the effects generated by SNHG16 knockdown

Subsequently, we explored the influence of miR-146a-5p and/or miR-7-5p inhibitors on the functions of HG-treated hRMECs with

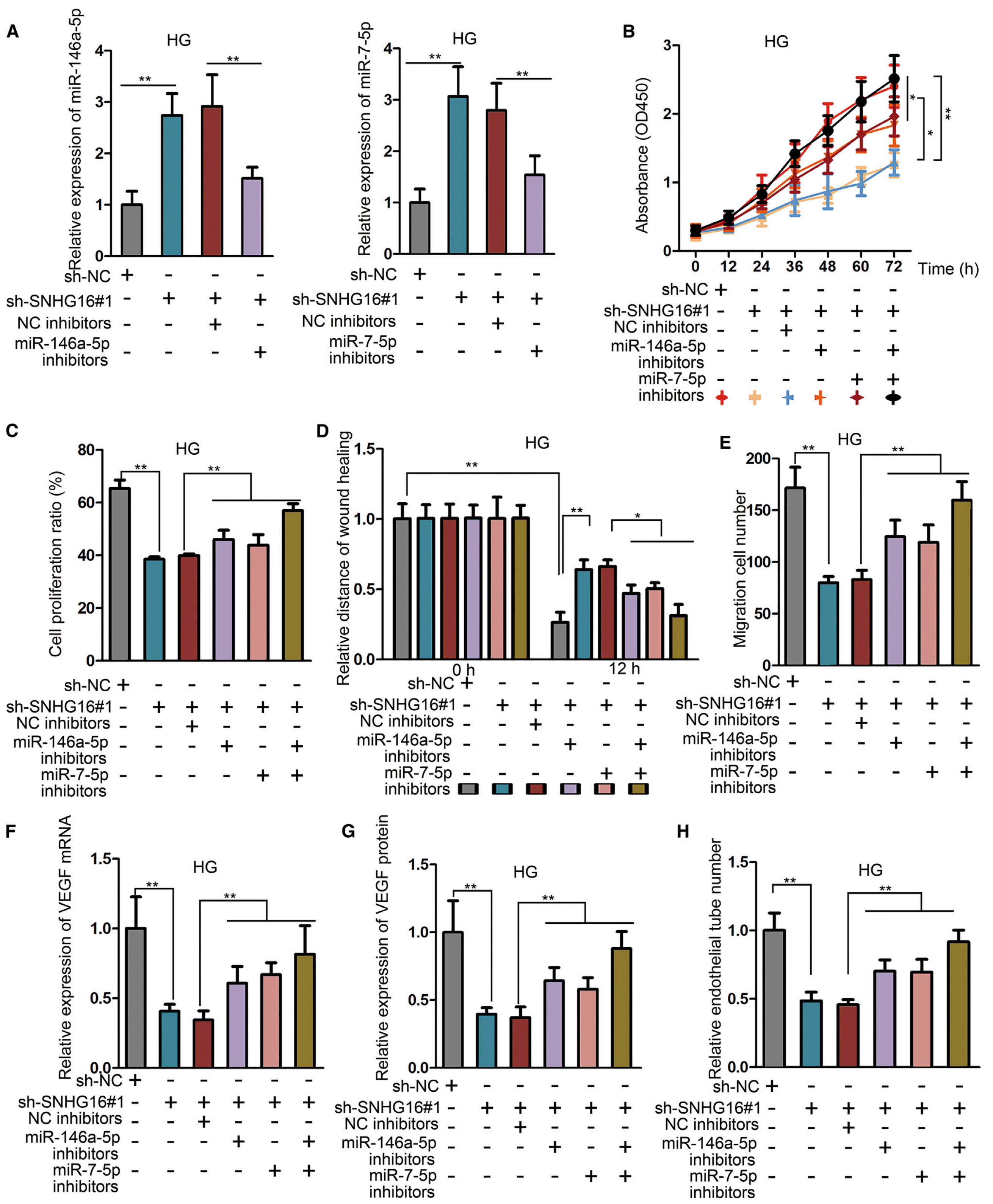

(legend on next page)

SNHG16 knockdown. At first, we found that the expression level of miR-146a-5p or miR-7-5p was decreased after transfection of corresponding inhibitors compared with NC inhibitors (Figure 5A). With the use of the CCK-8 assay and EdU assay, we observed that the inhibitory effect of SNHG16 knockdown on cell proliferation was partially rescued after transfection of miR-146a-5p or miR-7-5p inhibitors and was almost completely rescued by both of them (Figures 5B and 5C). Also, the wound-healing assay and Transwell assay showed that the inhibitory effect of SNHG16 knockdown on cell migration was partially rescued by miR-146a-5p or miR-7-5p inhibitors and was almost completely rescued by inhibition of both (Figures 5D and 5E). Then, we detected mRNA and protein levels of VEGF and found that VEGF level reduced by SNHG16 silencing was upregulated after miR-146a-5p and/or miR-7-5p knockdown (Figures 5F and 5G). Besides, angiogenesis impeded by SNHG16 knockdown was facilitated by transfection of miR-146a-5p and/or miR-7-5p inhibitors (Figure 5H). Taken together, the influence of SNHG16 knockdown in hRMECs was reversed by miR-146a-5p and miR-7-5p inhibitors, indicating that the biological function of SNHG16 was exerted via sponging miR-146a-5p and miR-7-5p.

Interleukin-1 receptor-associated kinase 1 (IRAK1) and insulin receptor substrate 1 (IRS1) are targeted, respectively, by miR-146a-5p and miR-7-5p

Our study continued to detect potential genes that were targeted by miR-146a-5p and miR-7-5p and could participate in hRMEC dysfunction via activating NF-κB and PI3K/AKT pathways. As previous studies indicated, miR-146a-5p could inhibit diabetes-induced defects in retinal endothelial cells via targeting several key molecules of the NF-κB pathway,[28,29] and miR-7-5p could reduce proliferation of retinal endothelial cells through targeting IRS1 and deactivating the PI3K/AKT pathway.[27] The levels of three pivotal genes targeted by miR-146a-5p were examined in response to SNHG16 knockdown in HG-treated hRMECs. We observed that only IRAK1 was significantly downregulated by SNHG16 knockdown (Figure 6A). We thus selected IRAK1 and IRS1 as the objects of our study. With the help of the ENCORI online database, we identified the binding sequences between miR-146a-5p and IRAK1 3′ UTR and between miR-7-5p and IRS1 3′ UTR (Figure 6B).

As shown by quantitative real-time PCR and western blot analyses, mRNA and protein levels of IRAK1 and IRS1 were significantly increased under the HG condition (Figures S3A and S3B). Besides, IRAK1 and IRS1 were upregulated by SNHG16 overexpression and downregulated by SNHG16 knockdown on both levels of mRNA and protein (Figures S3C and S3D). To determine the molecular interaction, we conducted a RIP assay, showing the enrichment of SNHG16, miR-146a-5p, miR-7-5p, and mRNAs of IRAK1 and IRS1 in Ago2 groups (Figure 6C). Then, an RNA pull-down assay illustrated that IRAK1 or IRS1 mRNAs directly interacted with WT biotinylated probes for miR-146a-5p or miR-7-5p (Figure 6D). Moreover, as shown by the luciferase reporter assay, the luciferase activity of WT IRAK1-3′ UTR and IRS1-3′ UTR reporters, not of reporters with Mut binding sites, was, respectively, reduced by addition of miR-146a-5p and miR-7-5p mimics and then both partially enhanced by SNHG16 overexpression (Figure 6E). These results elucidated that miR-146a-5p and miR-7-5p, respectively, target their downstream genes IRAK1 and IRS1 in hRMECs.

SNHG16 promotes hRMEC dysfunction via NF-κB pathway activation through IRAK1

To verify that the function of SNHG16 was exerted through the IRAK1 and NF-κB pathway, we transfected lentiviral vectors with shRNAs targeting IRAK1 into hRMECs after SNHG16 overexpression and found that the level of IRAK1, which had been enhanced by SNHG16 overexpression, was suppressed after transfecting shRNAs for IRAK1 (Figure 7A). The promotional effect of SNHG16 overexpression on cell proliferation was partially inhibited by transfection of IRAK1 shRNAs, as well as by addition of the NF-κB pathway inhibitor BAY 11-7082 (Figures 7B and 7C). Also, the enhanced cell migration was suppressed by both IRAK1 silencing and BAY 11-7082 addition (Figures 7D and 7E). After the upregulated VEGF level in SNHG16-overexpressed hRMECs was found to be reduced by both IRAK1 knockdown and NF-κB pathway inhibition (Figures 7F and 7G), we detected their influence on angiogenesis and discovered that both IRAK1 knockdown and BAY 11-7082 could impede angiogenesis facilitated by SNHG16 overexpression (Figure 7H).

Since BAY 11-7082 functions via inhibiting p-IκBα, we detected the protein levels of IRAK1, p-IκBα, and IκBα in response to IRAK1 knockdown or BAY 11-7082. We found that the IRAK1 protein level was affected by transfection of IRAK1 shRNAs but not by BAY 11-7082 treatment. Also, we discovered that p-IκBα enhanced by SNHG16 overexpression was reduced by both IRAK1 knockdown and BAY 11-7082 addition (Figure S4A). Furthermore, both IRAK1 knockdown and BAY 11-7082 addition could inhibit p65 nuclear translocation (Figure S4B) and attenuate NF-κB activity (Figure 7I), which had been enhanced by SNHG16 overexpression. To further strengthen the above results, we used another NF-κB inhibitor

Figure 5. miR-146a-5p and miR-7-5p inhibitors reverse the effects generated by SNHG16 knockdown

Experiments were conducted in cells treated with HG (25 mM) for 48 h. (A) Quantitative real-time PCR showed that the enhanced levels of miR-146a-5p and miR-7-5p in hRMECs after SNHG16 knockdown were suppressed by transfection of miR-146a-5p and miR-7-5p inhibitors, respectively. n = 3 in each group. (B and C) CCK-8 assay and EdU assay illustrated the effect of miR-146a-5p and/or miR-7-5p inhibitors on cell proliferation in hRMECs with SNHG16 knockdown. n = 3 in each group. (D and E) Wound-healing assay and Transwell assay illustrated the effect of miR-146a-5p and/or miR-7-5p inhibitors on cell migration in hRMECs with SNHG16 knockdown. n = 3 in each group. (F and G) Quantitative real-time PCR and ELISA showed the influence of miR-146a-5p and/or miR-7-5p inhibitors on VEGF level. n = 3 in each group. (H) Tube formation assay showed the influence of miR-146a-5p and/or miR-7-5p inhibitors on angiogenesis. n = 3 in each group. All data were acquired from three independent experiments and presented as the mean ± SD. *p < 0.05, **p < 0.01.

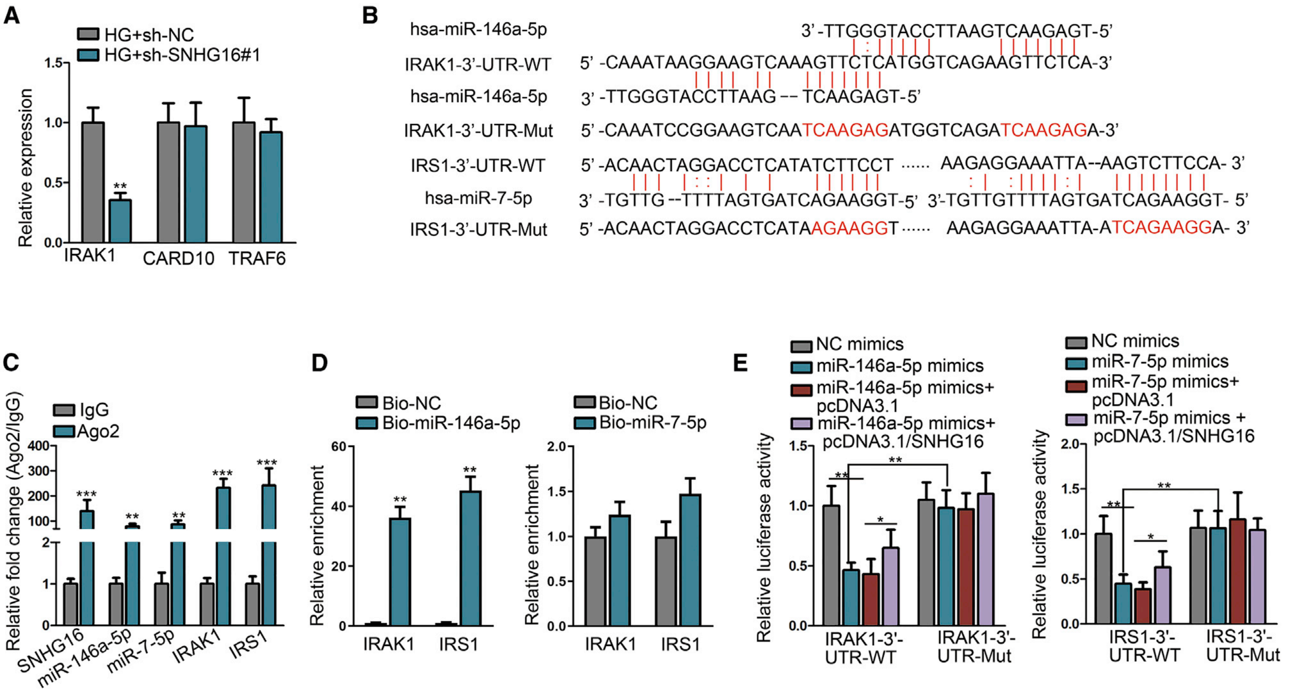

Figure 6. Interleukin-1 receptor-associated kinase 1 (IRAK1) and IRS1 are, respectively, targeted by miR-146a-5p and miR-7-5p

Experiments were conducted in cells treated with LG (5 mM) or HG (25 mM) for 48 h. (A) The levels of miR-146a-5p downstream genes, IRAK1, caspase recruitment domain family, member 10 (CARD10), and tumor necrosis factor receptor-associated factor 6 (TRAF6), were assessed using quantitative real-time PCR to detect the influence of SNHG16 knockdown on their expression. n = 3 in each group. (B) ENCORI prediction showed the binding sites of miR-146a-5p and miR-7-5p in the sequences of IRAK1 and IRS1 3′ UTRs. The sequences highlighted in red stand for the Mut binding sites for luciferase reporter assay. (C) RIP assay followed by quantitative real-time PCR demonstrated the enrichment of SNHG16, miR-146a-5p, miR-7-5p, and mRNAs of IRAK1 and IRS1 in RISCs. n = 3 in each group. (D) RNA pull-down assay followed by quantitative real-time PCR was performed to detect the level of IRAK1 or IRS1 mRNA interacting with miR-146a-5p or miR-7-5p. n = 3 in each group. (E) Luciferase reporter assay elucidated the interaction between IRAK1 or IRS1 mRNA and miR-146a-5p or miR-7-5p, as well as the competing effect of SNHG16 to sequester these miRNAs. n = 3 in each group. All data were acquired from three independent experiments and presented as the mean ± SD. *p < 0.05, **p < 0.01, ***p < 0.001.

(JSH-23) to repeat the above experiments. As illustrated in Figures S5A–S5H, JSH-23 had similar effects with BAY 11-7082. Meanwhile, the JSH-23 addition could enhance p-IκBα and inhibit p65 nuclear translocation (Figures S6A and S6B).

SNHG16 promotes hRMEC dysfunction via PI3K/AKT pathway activation through IRS1

Similarly, to verify that the function of SNHG16 was exerted through the IRS1 and PI3K/AKT pathway, shRNAs for IRS1 were transfected into hRMECs with SNHG16 overexpression, and the enhanced level of IRS1 was thus suppressed (Figure 8A). The promoted cell proliferation (Figures 8B and 8C) and migration (Figures 8D and 8E) was inhibited by both transfections of IRS1 shRNAs and addition of the PI3K/AKT pathway inhibitor LY294002. Also, both IRS1 knockdown and the PI3K/AKT pathway inhibition could reduce the VEGF level (Figures 8F and 8G) and angiogenesis (Figure 8H), which had been enhanced by SNHG16 overexpression. Furthermore, since LY294002 functions via inhibiting p-AKT, the protein levels of IRS1, PI3K, p-AKT, and AKT were measured in response to IRS1 knockdown or LY294002. We discovered that IRS1 and PI3K protein levels were affected by transfection of IRS1 shRNAs but not by LY294002 treatment, whereas p-AKT enhanced by SNHG16 overexpression was reduced by both IRS1 knockdown and LY294002 addition (Figure S7A). For further demonstration, we applied ZSTK474 to repeat the above experiments. As expected, ZSTK474 addition could attenuate the effects of SNHG16 overexpression on hRMEC functions (Figures S8A–S8G). The protein levels of IRS1 and PI3K enhanced by SNHG16 were decreased by IRS1 silencing but not affected by ZSTK474 treatment. The p-AKT enhanced by SNHG16 overexpression was reduced by both IRS1 knockdown and ZSTK474 addition (Figure S9A). In conclusion, the promotional effect of SNHG16 on hRMEC dysfunction was proven to be exerted via regulating IRAK1 and activating the NF-κB pathway and also via regulating IRS1 and activating the PI3K/AKT pathway.

DISCUSSION

Diabetes is associated with a variety of life-limiting complications, including macrovascular complications, such as cardiovascular disease and stroke, and microvascular complications, such as nephropathy and retinopathy.[30] As one of the most common microvascular complications of diabetes, DR leads to a rising number of visually impaired people worldwide.[31] Endothelial cells lining the inner

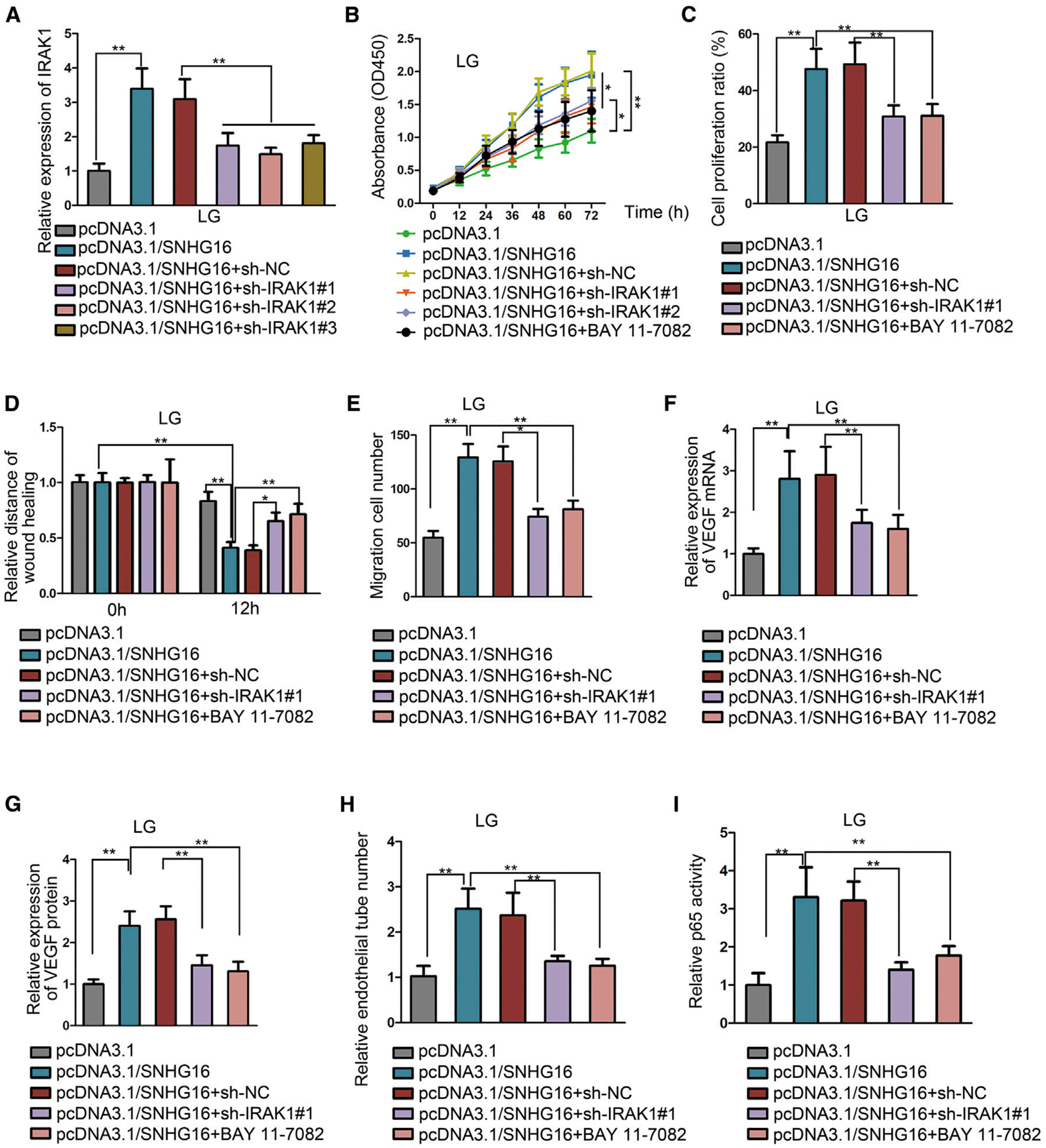

Figure 7. SNHG16 promotes hRMEC dysfunction via the NF-κB pathway activation through IRAK1

Experiments were conducted in cells treated with LG (5 mM) for 48 h. (A) Quantitative real-time PCR showed the change of IRAK1 level in SNHG16-overexpressed hRMECs by transfecting shRNAs for IRAK1. Two shRNAs with relatively higher knockdown capacity were selected. n = 3 in each group. (B and C) CCK-8 assay and EdU assay showed the effects of IRAK1 shRNAs and BAY 11-7082 on cell proliferation in SNHG16-overexpressed hRMECs. The shRNA with the highest knockdown capacity was adopted. n = 3 in each group. (D and E) Wound-healing assay and Transwell assay showed the effects of sh-IRAK1#1 transfection and BAY 11-7082 addition on cell migration in hRMECs after SNHG16 overexpression. n = 3 in each group. (F and G) Quantitative real-time PCR and ELISA showed the effects of sh-IRAK1#1 transfection and BAY 11-7082 addition on VEGF level in hRMECs after SNHG16 overexpression. n = 3 in each group. (H) Tube formation assay showed the effects of sh-IRAK1#1 transfection and BAY 11-7082 addition on angiogenesis in SNHG16-overexpressed hRMECs. n = 3 in each group. (I) ELISA-based NF-κB activity assay showed the change of NF-κB activity in response to IRAK1 knockdown or BAY 11-7082 treatment. n = 3 in each group. All data were acquired from three independent experiments and presented as the mean ± SD. $^{*}p < 0.05$, $^{**}p < 0.01$.

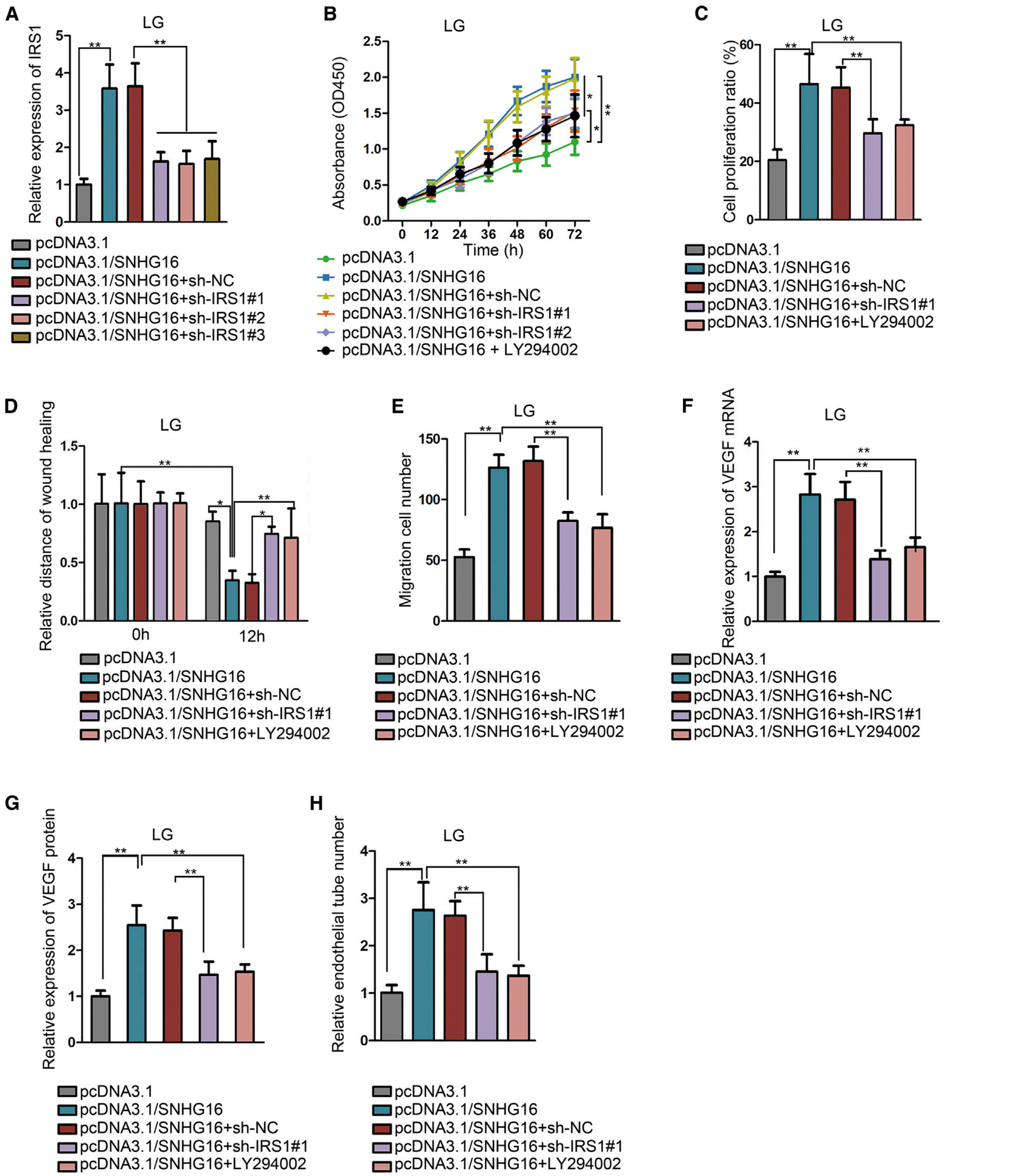

Figure 8. SNHG16 promotes hRMEC dysfunction via the PI3K/AKT pathway activation through IRS1

Experiments were conducted in cells treated with LG (5 mM) for 48 h. (A) Quantitative real-time PCR showed the change of IRS1 level in SNHG16-overexpressed hRMECs by transfecting shRNAs for IRS1. Two shRNAs with relatively higher knockdown capacity were selected. n = 3 in each group. (B and C) CCK-8 assay and EdU assay showed the effects of IRS1 shRNAs and LY294002 on cell proliferation in SNHG16-overexpressed hRMECs. The shRNA with the highest knockdown capacity was adopted. n = 3 in

(legend continued on next page)

surface of blood vessels play a critical role in maintaining vascular integrity and homeostasis.[32] After DR progresses from nonproliferative DR to the severe stage as proliferative DR, RMECs in the hyperglycemic state are also endowed with a proliferative phenotype and undergo abnormal proliferation, migration, and angiogenesis, which can gradually evolve into pathological neovascularization, vitreous hemorrhage, tractional retinal detachment, and loss of vision.[1,33] Our study herein discussed a potential mechanism that could contribute to these diabetes-related deleterious effects in hRMECs, providing new insight into DR treatment.

lncRNAs are characterized by accumulating studies as key regulators to modulate gene expression at various levels and thus play important roles in multifarious biological processes.[34] In recent years, lncRNAs are gaining attention, owing to the discovery of their participation in DR progression or prevention.[35] lncRNA SNHG16 has been studied in many cancers as an oncogene,[21–23] but its potential role in diabetic complications is still unclear. As previously reported, the promotional effect of SNHG16 on cell proliferation, migration, and angiogenesis has been identified in hemangioma endothelial cells.[17] Our study was aimed at evaluating the role of SNHG16 in modulating these abnormal functions of RMECs.

In order to imitate hyperglycemia-evoked retinal endothelial dysfunction, hRMECs were cultured under an HG condition, and the alterations of SNHG16 expression and subcellular distribution were detected. We discovered that SNHG16 was significantly upregulated after HG exposure in a dose-dependent pattern and in a time-dependent pattern, indicating that the SNHG16 level could be related with the development of DR. Thus, we hypothesized that SNHG16 could contribute to the proliferative DR-related abnormalities of endothelial cells. Therefore, we assessed the influence of SNHG16 overexpression or knockdown and discovered the promotional effect of SNHG16 on cell proliferation and migration. Since VEGF has been demonstrated as a pivotal factor in DR progression through stimulating vascular endothelial cell proliferation and migration via various signaling pathways, thus facilitating neovascularization,[18,33,36] the influence of SNHG16 on VEGF level and angiogenic activity of hRMECs was also evaluated, verifying the potentiality of SNHG16 in facilitating hRMEC dysfunction. Importantly, we uncovered the effect of SNHG16 on the HIF-α/VEGF axis.

Subsequently, to investigate the mechanism of SNHG16 in hRMECs, we further detected the transactivation levels of various important transcription factors after SNHG16 knockdown or overexpression using Cignal Reporter Assay based on dual-luciferase technology. We found that the transactivation levels of NF-κB and PI3K/AKT signaling were remarkably influenced by differential expression of SNHG16. According to many reports, NF-κB and PI3K/AKT pathways are both important signaling pathways participating in DR progression and pathogenesis, dysfunction of endothelial cells, and VEGF level.[37–40] In our study, the promotional influences of SNHG16 on p-IκBα, p65 nuclear translocation, and p65 activity, as well as PI3K expression and p-AKT, were detected, illustrating the participation of SNHG16 in regulating NF-κB and PI3K/AKT pathways in hRMECs.

The regulatory network of ceRNA has been widely demonstrated. Previously, many convincing investigations indicated the ceRNA role of SNHG16 in various diseases.[21–23] Based on the cytoplasmic location of SNHG16 in hRMECs, we considered that the possible mechanism of SNHG16 was sponging critical miRNAs, which could affect the expression of downstream genes involved in NF-κB and PI3K/AKT pathways. With the help of a bioinformatics database, among the candidate miRNAs to be directly targeted by SNHG16, miR-146a-5p and miR-7-5p were chosen for the reason that they could, respectively, inhibit NF-κB and PI3K/AKT pathways, thus attenuating abnormalities in retinal endothelial cells[25–27] and also in many other cases.[41–44] We thus validated the regulatory effects of the HG condition and SNHG16 level on the expression of miR-146a-5p or miR-7-5p and examined their molecular interactions by performing RIP, RNA pull-down, and luciferase reporter assays. Moreover, the regulatory effects induced by SNHG16 knockdown were partially reversed by transfecting either miR-146a-5p or miR-7-5p inhibitors and were almost completely rescued after transfection of both inhibitors, suggesting that SNHG16 functioned by acting as a molecular sponge of both miR-146a-5p and miR-7-5p in hRMECs.

Furthermore, miRNA downstream genes were selected by examining expression change in response to SNHG16 knockdown. We chose IRAK1 and IRS1, which were, respectively, targeted by miR-146a-5p or miR-7-5p, for our investigation. The activating effects of IRAK1 on the NF-κB pathway and of IRS1 on the PI3K/AKT pathway were studied in retinal endothelial cells[27–29] and also in many other cases.[45–47] Then the molecular interaction regarding the ceRNA network of SNHG16/miR-146a-5p/IRAK1 or SNHG16/miR-7-5p/IRS1 was determined. In our study, both HG condition and SNHG16 upregulation were found to increase the expression of IRAK1 and IRS1, activate NF-κB and PI3K/AKT signaling pathways, and lead to hRMEC dysfunction. Besides, the influences of SNHG16 overexpression were attenuated by knockdown of IRAK1 or IRS1 or by addition of the NF-κB or PI3K/AKT pathway inhibitor, indicating the mechanism underlying the effect of SNHG16 on hRMEC dysfunction.

In conclusion, our study elucidated the function and mechanism of lncRNA SNHG16 in regulating diabetes-associated retinal

each group. (D and E) Wound-healing assay and Transwell assay showed the effects of sh-IRS1#1 transfection and LY294002 addition on cell migration in hRMECs after SNHG16 overexpression. n = 3 in each group. (F and G) Quantitative real-time PCR and ELISA showed the effects of sh-IRS1#1 transfection and LY294002 addition on VEGF level in hRMECs after SNHG16 overexpression. n = 3 in each group. (H) Tube formation assay showed the effects of sh-IRS1#1 transfection and LY294002 addition on angiogenesis in SNHG16-overexpressed hRMECs. n = 3 in each group. All data were acquired from three independent experiments and presented as the mean ± SD. $^{*}p < 0.05$, $^{**}p < 0.01$.

endothelial cell dysfunction. Upregulation of SNHG16 in HG-stimulated hRMECs facilitates proliferative DR-related abnormalities in cell proliferation, migration, and angiogenesis through regulating miR-146a-5p/IRAK1 and miR-7-5p/IRS1 to activate NF-κB and PI3K/AKT signaling pathways. This research may pave the way for developing improved therapeutics in treating diabetes-related microvascular complications. Nevertheless, a limitation of this study needs to be discussed. According to previous studies, upregulation of lncRNAs can be induced by their upstream regulators, such as transcriptional activators. Recently, SNHG16 has been reported to be transcriptionally activated by CCCTC-binding factor (CTCF)[48] and transcription factor AP-2 alpha (TFAP2A)[49] in human cancers. Therefore, we will explore the mechanism that led to the upregulation of SNHG16 in HG treatment in our future study.

MATERIALS AND METHODS

Cell culture and treatment

hRMECs (HEC09) for our study were bought from Neuromics (Edina, MN, USA) and cultivated using EGM-2 Endothelial Cell Growth Medium-2 BulletKit (Lonza, Basel, Switzerland), comprised of endothelial basal medium (EBM-2; Lonza) and EGM-2 SingleQuots Supplement Pack (Lonza). Cells were allowed to grow in T75 flasks under 95% air and 5% CO_2 at 37°C in a CO_2 incubator (Thermo Scientific, Glen Burnie, MD, USA). The confluent cells were collected after 48 h of culturing with HG (25 mM D-glucose; termed HG group), LG (5 mM D-glucose; termed LG group), or 25 mM L-glucose as Osm (Osm group), unless otherwise specified. For pathway inhibition assay, cell cultures were supplemented with NF-κB pathway inhibitor BAY 11-7082 (2 μM) or PI3K/AKT pathway inhibitor LY294002 (10 μM). D-glucose and L-glucose were purchased from Sigma-Aldrich (St. Louis, MO, USA). BAY 11-7082 and LY294002 were purchased from MedChemExpress (Monmouth Junction, NJ, USA).

Cell transfection

hRMECs at 70%–80% confluence were reaped and transfected with indicated plasmids for 48 h using Lipofectamine 2000 (Invitrogen, Carlsbad, CA, USA). Overexpression plasmid pcDNA3.1/SNHG16 and pcDNA3.1 negative control (NC), as well as triple shRNAs against SNHG16 (sh-SNHG16#1/2/3), IRAK1 (sh-IRAK1#1/2/3), IRS1 (sh-IRS1#1/2/3), and NC shRNAs (sh-NC), were all procured from GenePharma (Shanghai, China). miRNA mimics and inhibitors (miR-146a-5p mimics/inhibitors, miR-7-5p mimics/inhibitors, and NC mimics/inhibitors) were all purchased from GeneChem (Shanghai, China). The shRNA sequences were listed in Table S2.

RNA extraction and quantitative real-time PCR analysis

Total RNAs were extracted from hRMECs with the help of a TRIzol reagent (Invitrogen) and then treated with DNase I (Invitrogen) for removing DNA contaminates. After quantification using a Nanodrop 2000 spectrophotometer (Thermo Scientific), complementary DNAs (cDNAs) were synthesized using the PrimeScript RT reagent Kit (Takara, Kusatsu, Japan), and quantitative real-time PCR was then implemented using the SYBR Premix ExTaqII kit (Takara) on a CFX96 Touch Real-Time PCR Detection System (Bio-Rad, Hercules, CA, USA). Glyceraldehyde 3-phosphate dehydrogenase (GAPDH) and U6 served as loading controls. After cycle threshold value was determined, data were analyzed through the $2^{-\Delta\Delta Ct}$ method. The primers were purchased from GenePharma, and their sequences are listed in Table S1.

Subcellular fractionation assay

The fractions of cytoplasm and nucleus were isolated from hRMECs in light of the instruction manual of the PARIS Kit (Invitrogen). Briefly, 1×10^6 hRMECs were initially rinsed in ice-cold phosphate-buffered saline (PBS; Sigma-Aldrich) and then treated in turn with cell fractionation buffer and cell disruption buffer. Quantitative real-time PCR was applied to detect the levels of GAPDH, SNHG16, and U6 in cytoplasmic or nuclear fraction.

Western blot analysis

Total proteins from hRMECs were isolated using radioimmunoprecipitation assay (RIPA) lysis buffer (Solarbio, Beijing, China) containing protease inhibitors. Protein samples were separated using 10% sodium dodecyl sulfate-polyacrylamide gel electrophoresis (SDS-PAGE) gel (Beyotime, Shanghai, China) and transferred onto polyvinylidene difluoride (PVDF) membranes (Millipore, Burlington, MA, USA). After sealed with 5% nonfat milk for 2 h, PVDF membranes were incubated with specific primary antibodies at 4°C overnight. Horseradish peroxidase (HRP)-conjugated secondary antibodies (1:20,000 dilutions; ab205718; Abcam, Cambridge, UK) were subsequently applied. Enhanced chemiluminescence (ECL) detection system (Thermo Scientific) was used for detection of immunoblots. ImageJ software (NIH, Bethesda, MD, USA) was used to analyze protein band grayscale normalized to β-actin. The primary antibodies are listed below: p-IκBα (1:1,000; #9246; Cell Signaling Technology, Danvers, MA, USA), IκBα (1:1,000; #4812; Cell Signaling Technology), p65 (0.5 μg/mL; ab16502; Abcam), PI3K (1:400; sc-365290; Santa Cruz Biotechnology, Santa Cruz, CA, USA), p-AKT (1:2,000; #4060; Cell Signaling Technology), AKT (1:1,000; #9272; Cell Signaling Technology), IRAK1 (1:1,000; ab238; Abcam), IRS1 (1:400; sc-8038; Santa Cruz Biotechnology), and β-actin (1:1,000; ab69512; Abcam).

ELISA

After 48 h of cell transfection, the culture medium of hRMECs was collected from each group and preserved at −80°C. VEGF level in culture medium was measured using the Human VEGF Quantikine ELISA Kit (R&D Systems, Minneapolis, MN, USA) based on the user guide. Optical density at 450 nm (OD450) was detected by the ELx808 Absorbance Microplate Reader (BioTek, Winooski, VT, USA) and considered as the measurement of relative VEGF level.

Cell proliferation assay

Cell proliferation was assessed through the CCK-8 assay and EdU assay. For the CCK-8 assay, after 0, 24, 48, 72, and 96 h of cell transfection, hRMECs in 96-well plates were harvested after 2 h of incubation with 10 μL CCK-8 reagent (Dojindo Laboratories, Kumamoto, Japan). Cell viability was monitored by measuring OD450 using a

microplate reader (BioTek). For the EdU assay, transfected hRMECs in 96-well plates were prepared for 3 h of incubation using the Cell-Light EdU Apollo488 *In Vitro* Kit (RiboBio, Guangzhou, China). EdU labeling medium was applied at a concentration of 50 μM EdU. After they were fixed and rinsed, cells were treated with 1 × Apollo 488 and Hoechst solution (5 μg/mL, 100 μL) in sequence for visualization under a fluorescence microscope (Olympus, Tokyo, Japan).

Wound-healing assay

hRMECs were first placed into 96-well plates (5×10^4 cells/well) for overnight incubation until confluence was about 90%. The cell layer was scratched by a sterile pipette tip to prepare the wound. After they were washed with PBS, hRMECs were incubated for 12 h. The image of wound healing was visualized through optical microscopy (Nikon, Tokyo, Japan) and analyzed using ImageJ software for wound-healing distance quantification.

Transwell migration assay

The migration ability of hRMECs was evaluated using the Corning Transwell Multiple Well Plate (Fisher Scientific, Pittsburgh, PA, USA). The underneath of membrane insert was previously coated with 10 μg/mL fibronectin (Fisher Scientific). Then the Transwell units were maintained at 4°C overnight and sealed with 1% bovine serum albumin (BSA; Fisher Scientific) at 37°C for 1 h. Next, 3×10^4 hRMECs were added into the top chamber and incubated at 37°C for 6 h. Finally, the migrated cells in the bottom chamber were immobilized and dyed with crystal violet (Sigma-Aldrich), and the migration cell number was calculated with the aid of an optical microscope (Nikon).

Tube formation assay

The angiogenesis ability was detected using the *In Vitro* Angiogenesis Assay Kit (Sigma-Aldrich), as instructed by the user guide. In brief, hRMECs were cultured in 96-well plates (1.5×10^4 cells/well) coated with ECMatrix solid gel at 37°C. After 24 h of incubation, the relative endothelial tube number was determined using an inverse phase contrast light microscope (Carl Zeiss Meditec AG, Jena, Germany) and ImageJ software.

FISH assay

A specific FISH probe for SNHG16 was synthesized by RiboBio. The probe sequences were listed in Table S2. hRMECs were incubated with probe hybridization at 37°C overnight, immobilized with 4% formaldehyde, and washed with PBS. Cell nucleus was counterstained with Hoechst 33342 (Fanbo, Beijing, China). Images were captured using a fluorescence microscope (Olympus). ImageJ software was adopted for FISH data quantification.

RIP assay

A RIP assay was implemented using anti-Ago2 antibodies (ab32381; Abcam) or NC anti-human IgG (ab6715; Abcam), according to the guideline of EZ-Magna RIP Kit (Millipore). Cell lysate was prepared with RIP lysis buffer containing protease inhibitors and RNase inhibitors. Then 100 μL lysate was coincubated with magnetic beads and 5 μg antibodies in 50 μL RIP buffer. After proteinase K treatment and centrifugation, quantitative real-time PCR was conducted for analyzing immunoprecipitated RNAs.

RNA pull-down assay

Biotin RNA Labeling Mix (Roche, Basel, Switzerland) was utilized to construct WT or Mut biotinylated oligonucleotides. Briefly, cell lysate from hRMECs was collected and coincubated with biotinylated RNA probes and streptavidin agarose beads (Life Technologies, Gaithersburg, MD, USA) on ice for 1 h. The levels of specific RNAs were measured using quantitative real-time PCR after purification.

Dual-luciferase reporter assay

To detect the molecular interaction between miRNAs and their target transcripts, luciferase reporter assay was performed via co-transfection of luciferase reporters and the indicated transfection plasmids in hRMECs for 48 h. pmirGLO Dual-Luciferase miRNA Target Expression Vectors (Promega, Madison, WI, USA) were utilized to construct luciferase reporters containing specific WT or Mut binding sites. Mutation was established using the QuikChange Site-Directed Mutagenesis Kit (Stratagene, La Jolla, CA, USA). Dual-Luciferase Reporter Assay System (Promega) was finally applied to examine relative luciferase activity (the ratio of firefly/Renilla luciferase activity).

Cignal Reporter Assay

The Cignal Reporter Assay Kit was acquired from QIAGEN (Germantown, MD, USA) for determining the transactivation of transcription factors based on dual-luciferase reporter technology. According to the protocol, in brief, hRMECs were cotransfected with transcription factor-responsive reporters and the indicated transfection plasmids. After cell transfection, cell lysate was obtained using cell lysis buffer (Promega). Luciferase activities of firefly and Renilla were severely tested by the Dual-Luciferase Reporter Assay System (Promega). Firefly/Renilla activity ratios were normalized by those from NC transfections to obtain relative luciferase units, representing the signaling activities of specific pathways.

NF-κB activity assay

The ELISA-based TransAM NF-κB Family Kit was acquired from Active Motif (Carlsbad, CA, USA). After the nuclear fraction of hRMECs was isolated, the oligonucleotides coated on a plate containing a NF-κB response element sequence (5′-GGGACTTTCC-3′) were used to sequester active NF-κB. Then antibodies against p65, a main regulatory component of the NF-κB family, were applied, followed by treatment with HRP-conjugated secondary antibodies. The absorbance at 450 nm was detect using a microplate reader (BioTek).

Statistical analysis

Statistical analysis of this study was conducted using GraphPad Prism 6.0 software (La Jolla, CA, USA). Significance of differences between two groups was analyzed by two-tailed and unpaired Student's t test, whereas the differences among multiple groups were analyzed by one-way or two-way analysis of variance (ANOVA). A probability value of

$p < 0.05$ was considered to be statistically significant. All quantitative data were given as the mean ± standard deviation (SD) from independent bio-triplications.

SUPPLEMENTAL INFORMATION

Supplemental Information can be found online at https://doi.org/10.1016/j.omtn.2021.01.035.

ACKNOWLEDGMENTS

We sincerely thank all people involved. This study was supported by Hubei Natural Science Foundation (2019CFB499), Scientific Research Fund of Union Hospital Affiliated to Tongji Medical College of Huazhong University of Science and Technology (02.03.2018-244), and National Natural Science Foundation of China (81900432).

AUTHOR CONTRIBUTIONS

Project administration, conceptualization, software, formal analysis, data curation, writing – review & editing, and supervision, F.C. and H.J.; investigation and methodology, Y.L.; writing – original draft preparation, Q.L. and C.Y. All authors have read and approved the final manuscript.

DECLARATION OF INTERESTS

The authors declare no competing interests.

REFERENCES

1. Wong, T.Y., Cheung, C.M., Larsen, M., Sharma, S., and Simó, R. (2016). Diabetic retinopathy. Nat. Rev. Dis. Primers *2*, 16012.
2. Klein, R., Lee, K.E., Gangnon, R.E., and Klein, B.E. (2010). The 25-year incidence of visual impairment in type 1 diabetes mellitus the wisconsin epidemiologic study of diabetic retinopathy. Ophthalmology *117*, 63–70.
3. Yau, J.W., Rogers, S.L., Kawasaki, R., Lamoureux, E.L., Kowalski, J.W., Bek, T., Chen, S.J., Dekker, J.M., Fletcher, A., Grauslund, J., et al.; Meta-Analysis for Eye Disease (META-EYE) Study Group (2012). Global prevalence and major risk factors of diabetic retinopathy. Diabetes Care *35*, 556–564.
4. Stitt, A.W., Curtis, T.M., Chen, M., Medina, R.J., McKay, G.J., Jenkins, A., Gardiner, T.A., Lyons, T.J., Hammes, H.P., Simó, R., and Lois, N. (2016). The progress in understanding and treatment of diabetic retinopathy. Prog. Retin. Eye Res. *51*, 156–186.
5. Bharadwaj, A.S., Appukuttan, B., Wilmarth, P.A., Pan, Y., Stempel, A.J., Chipps, T.J., Benedetti, E.E., Zamora, D.O., Choi, D., David, L.L., and Smith, J.R. (2013). Role of the retinal vascular endothelial cell in ocular disease. Prog. Retin. Eye Res. *32*, 102–180.
6. Ding, J., and Wong, T.Y. (2012). Current epidemiology of diabetic retinopathy and diabetic macular edema. Curr. Diab. Rep. *12*, 346–354.
7. Ceriello, A. (2010). Point: postprandial glucose levels are a clinically important treatment target. Diabetes Care *33*, 1905–1907.
8. Antonetti, D.A., Barber, A.J., Bronson, S.K., Freeman, W.M., Gardner, T.W., Jefferson, L.S., Kester, M., Kimball, S.R., Krady, J.K., LaNoue, K.F., et al.; JDRF Diabetic Retinopathy Center Group (2006). Diabetic retinopathy: seeing beyond glucose-induced microvascular disease. Diabetes *55*, 2401–2411.
9. Fatica, A., and Bozzoni, I. (2014). Long non-coding RNAs: new players in cell differentiation and development. Nat. Rev. Genet. *15*, 7–21.
10. Rinn, J.L., and Chang, H.Y. (2012). Genome regulation by long noncoding RNAs. Annu. Rev. Biochem. *81*, 145–166.
11. Wahlestedt, C. (2013). Targeting long non-coding RNA to therapeutically upregulate gene expression. Nat. Rev. Drug Discov. *12*, 433–446.
12. Viereck, J., Kumarswamy, R., and Thum, T. (2015). Long noncoding RNAs as inducers and terminators of vascular development. Circulation *131*, 1236–1238.
13. Jaé, N., and Dimmeler, S. (2015). Long noncoding RNAs in diabetic retinopathy. Circ. Res. *116*, 1104–1106.
14. Yan, B., Yao, J., Liu, J.Y., Li, X.M., Wang, X.Q., Li, Y.J., Tao, Z.F., Song, Y.C., Chen, Q., and Jiang, Q. (2015). lncRNA-MIAT regulates microvascular dysfunction by functioning as a competing endogenous RNA. Circ. Res. *116*, 1143–1156.
15. Qiu, G.Z., Tian, W., Fu, H.T., Li, C.P., and Liu, B. (2016). Long noncoding RNA-MEG3 is involved in diabetes mellitus-related microvascular dysfunction. Biochem. Biophys. Res. Commun. *471*, 135–141.
16. Thomas, A.A., Biswas, S., Feng, B., Chen, S., Gonder, J., and Chakrabarti, S. (2019). lncRNA H19 prevents endothelial-mesenchymal transition in diabetic retinopathy. Diabetologia *62*, 517–530.
17. Zhao, W., Fu, H., Zhang, S., Sun, S., and Liu, Y. (2018). LncRNA SNHG16 drives proliferation, migration, and invasion of hemangioma endothelial cell through modulation of miR-520d-3p/STAT3 axis. Cancer Med. *7*, 3311–3320.
18. Neufeld, G., Cohen, T., Gengrinovitch, S., and Poltorak, Z. (1999). Vascular endothelial growth factor (VEGF) and its receptors. FASEB J. *13*, 9–22.
19. Salmena, L., Poliseno, L., Tay, Y., Kats, L., and Pandolfi, P.P. (2011). A ceRNA hypothesis: the Rosetta Stone of a hidden RNA language? Cell *146*, 353–358.
20. Karreth, F.A., and Pandolfi, P.P. (2013). ceRNA cross-talk in cancer: when ce-bling rivalries go awry. Cancer Discov. *3*, 1113–1121.
21. Cai, C., Huo, Q., Wang, X., Chen, B., and Yang, Q. (2017). SNHG16 contributes to breast cancer cell migration by competitively binding miR-98 with E2F5. Biochem. Biophys. Res. Commun. *485*, 272–278.
22. Zhu, H., Zeng, Y., Zhou, C.C., and Ye, W. (2018). SNHG16/miR-216-5p/ZEB1 signal pathway contributes to the tumorigenesis of cervical cancer cells. Arch. Biochem. Biophys. *637*, 1–8.
23. Feng, F., Chen, A., Huang, J., Xia, Q., Chen, Y., and Jin, X. (2018). Long noncoding RNA SNHG16 contributes to the development of bladder cancer via regulating miR-98/STAT3/Wnt/β-catenin pathway axis. J. Cell. Biochem. *119*, 9408–9418.
24. Li, J.H., Liu, S., Zhou, H., Qu, L.H., and Yang, J.H. (2014). starBase v2.0: decoding miRNA-ceRNA, miRNA-ncRNA and protein-RNA interaction networks from large-scale CLIP-Seq data. Nucleic Acids Res. *42*, D92–D97.
25. Wang, Q., Bozack, S.N., Yan, Y., Boulton, M.E., Grant, M.B., and Busik, J.V. (2014). Regulation of retinal inflammation by rhythmic expression of MiR-146a in diabetic retina. Invest. Ophthalmol. Vis. Sci. *55*, 3986–3994.
26. Ye, E.A., and Steinle, J.J. (2016). miR-146a Attenuates Inflammatory Pathways Mediated by TLR4/NF-κB and TNFα to Protect Primary Human Retinal Microvascular Endothelial Cells Grown in High Glucose. Mediators Inflamm. *2016*, 3958453.
27. Cao, Y.L., Liu, D.J., and Zhang, H.G. (2018). MiR-7 regulates the PI3K/AKT/VEGF pathway of retinal capillary endothelial cell and retinal pericytes in diabetic rat model through IRS-1 and inhibits cell proliferation. Eur. Rev. Med. Pharmacol. Sci. *22*, 4427–4430.
28. Cowan, C., Muraleedharan, C.K., O'Donnell, J.J., 3rd, Singh, P.K., Lum, H., Kumar, A., and Xu, S. (2014). MicroRNA-146 inhibits thrombin-induced NF-κB activation and subsequent inflammatory responses in human retinal endothelial cells. Invest. Ophthalmol. Vis. Sci. *55*, 4944–4951.
29. Zhuang, P., Muraleedharan, C.K., and Xu, S. (2017). Intraocular Delivery of miR-146 Inhibits Diabetes-Induced Retinal Functional Defects in Diabetic Rat Model. Invest. Ophthalmol. Vis. Sci. *58*, 1646–1655.
30. Forbes, J.M., and Cooper, M.E. (2013). Mechanisms of diabetic complications. Physiol. Rev. *93*, 137–188.
31. Leasher, J.L., Bourne, R.R., Flaxman, S.R., Jonas, J.B., Keeffe, J., Naidoo, K., Pesudovs, K., Price, H., White, R.A., Wong, T.Y., et al.; Vision Loss Expert Group of the Global Burden of Disease Study (2016). Global Estimates on the Number of People Blind or Visually Impaired by Diabetic Retinopathy: A Meta-analysis From 1990 to 2010. Diabetes Care *39*, 1643–1649.
32. Dejana, E. (2004). Endothelial cell-cell junctions: happy together. Nat. Rev. Mol. Cell Biol. *5*, 261–270.
33. Simó, R., Carrasco, E., García-Ramírez, M., and Hernández, C. (2006). Angiogenic and antiangiogenic factors in proliferative diabetic retinopathy. Curr. Diabetes Rev. *2*, 71–98.

34. Wang, K.C., and Chang, H.Y. (2011). Molecular mechanisms of long noncoding RNAs. Mol. Cell *43*, 904–914.

35. Gong, Q., and Su, G. (2017). Roles of miRNAs and long noncoding RNAs in the progression of diabetic retinopathy. Biosci. Rep. *37*, BSR20171157.

36. Behl, T., and Kotwani, A. (2015). Exploring the various aspects of the pathological role of vascular endothelial growth factor (VEGF) in diabetic retinopathy. Pharmacol. Res. *99*, 137–148.

37. Kowluru, R.A., Koppolu, P., Chakrabarti, S., and Chen, S. (2003). Diabetes-induced activation of nuclear transcriptional factor in the retina, and its inhibition by antioxidants. Free Radic. Res. *37*, 1169–1180.

38. Choudhuri, S., Chowdhury, I.H., Das, S., Dutta, D., Saha, A., Sarkar, R., Mandal, L.K., Mukherjee, S., and Bhattacharya, B. (2015). Role of NF-κB activation and VEGF gene polymorphisms in VEGF up regulation in non-proliferative and proliferative diabetic retinopathy. Mol. Cell. Biochem. *405*, 265–279.

39. Abid, M.R., Guo, S., Minami, T., Spokes, K.C., Ueki, K., Skurk, C., Walsh, K., and Aird, W.C. (2004). Vascular endothelial growth factor activates PI3K/Akt/forkhead signaling in endothelial cells. Arterioscler. Thromb. Vasc. Biol. *24*, 294–300.

40. Cui, J., Gong, R., Hu, S., Cai, L., and Chen, L. (2018). Gambogic acid ameliorates diabetes-induced proliferative retinopathy through inhibition of the HIF-1α/VEGF expression via targeting PI3K/AKT pathway. Life Sci. *192*, 293–303.

41. Cheng, H.S., Sivachandran, N., Lau, A., Boudreau, E., Zhao, J.L., Baltimore, D., Delgado-Olguin, P., Cybulsky, M.I., and Fish, J.E. (2013). MicroRNA-146 represses endothelial activation by inhibiting pro-inflammatory pathways. EMBO Mol. Med. *5*, 1017–1034.

42. Liu, R., Liu, C., Chen, D., Yang, W.H., Liu, X., Liu, C.G., Dugas, C.M., Tang, F., Zheng, P., Liu, Y., and Wang, L. (2015). FOXP3 Controls an miR-146/NF-κB Negative Feedback Loop That Inhibits Apoptosis in Breast Cancer Cells. Cancer Res. *75*, 1703–1713.

43. Fang, Y., Xue, J.L., Shen, Q., Chen, J., and Tian, L. (2012). MicroRNA-7 inhibits tumor growth and metastasis by targeting the phosphoinositide 3-kinase/Akt pathway in hepatocellular carcinoma. Hepatology *55*, 1852–1862.

44. Liu, Z., Jiang, Z., Huang, J., Huang, S., Li, Y., Yu, S., Yu, S., and Liu, X. (2014). miR-7 inhibits glioblastoma growth by simultaneously interfering with the PI3K/ATK and Raf/MEK/ERK pathways. Int. J. Oncol. *44*, 1571–1580.

45. Wee, Z.N., Yatim, S.M., Kohlbauer, V.K., Feng, M., Goh, J.Y., Bao, Y., Lee, P.L., Zhang, S., Wang, P.P., Lim, E., et al. (2015). IRAK1 is a therapeutic target that drives breast cancer metastasis and resistance to paclitaxel. Nat. Commun. *6*, 8746.

46. Saltiel, A.R., and Pessin, J.E. (2002). Insulin signaling pathways in time and space. Trends Cell Biol. *12*, 65–71.

47. Mi, L., Chen, Y., Zheng, X., Li, Y., Zhang, Q., Mo, D., and Yang, G. (2015). MicroRNA-139-5p Suppresses 3T3-L1 Preadipocyte Differentiation Through Notch and IRS1/PI3K/Akt Insulin Signaling Pathways. J. Cell. Biochem. *116*, 1195–1204.

48. Wang, D., Lin, B., Zhang, W., and Wang, X. (2020). Up-regulation of SNHG16 induced by CTCF accelerates cardiac hypertrophy by targeting miR-182-5p/IGF1 axis. Cell Biol. Int. *44*, 1426–1435.

49. Zhang, G., Ma, A., Jin, Y., Pan, G., and Wang, C. (2019). LncRNA SNHG16 induced by TFAP2A modulates glycolysis and proliferation of endometrial carcinoma through miR-490-3p/HK2 axis. Am. J. Transl. Res. *11*, 7137–7145.

OMTN, Volume *24*

Supplemental information

Upregulation of long non-coding RNA SNHG16 promotes diabetes-related RMEC dysfunction via activating NF-κB and PI3K/AKT pathways

Fei Cai, Huanzong Jiang, Yan Li, Qin Li, and Chao Yang

Figure S1 HG enhances hRMEC viability and proliferation. (A) CCK-8 assay was applied to evaluate the cell viability after treated with HG at 0h, 12h and 48h. N=3 in each group. (B) Cell proliferation ratio of each group (0h, 12h and 48h) was illustrated through calculating the percentage of EdU positive cells using EdU assay. N=3 in each group. (C) The morphology of hRMECs treated with LG or HG was observed under a microscope. (D) The ROS level in HG-treated hRMECs after SNHG16 silencing. (E) Apoptosis-related proteins were detected in hRMECs after indicated transfections. (F) The levels of HIF-1α and VEGF were measured in hRMECs after indicated transfections. All data were acquired from three independent experiments and presented as the mean ± SD. *$p < 0.05$, **$p < 0.01$.

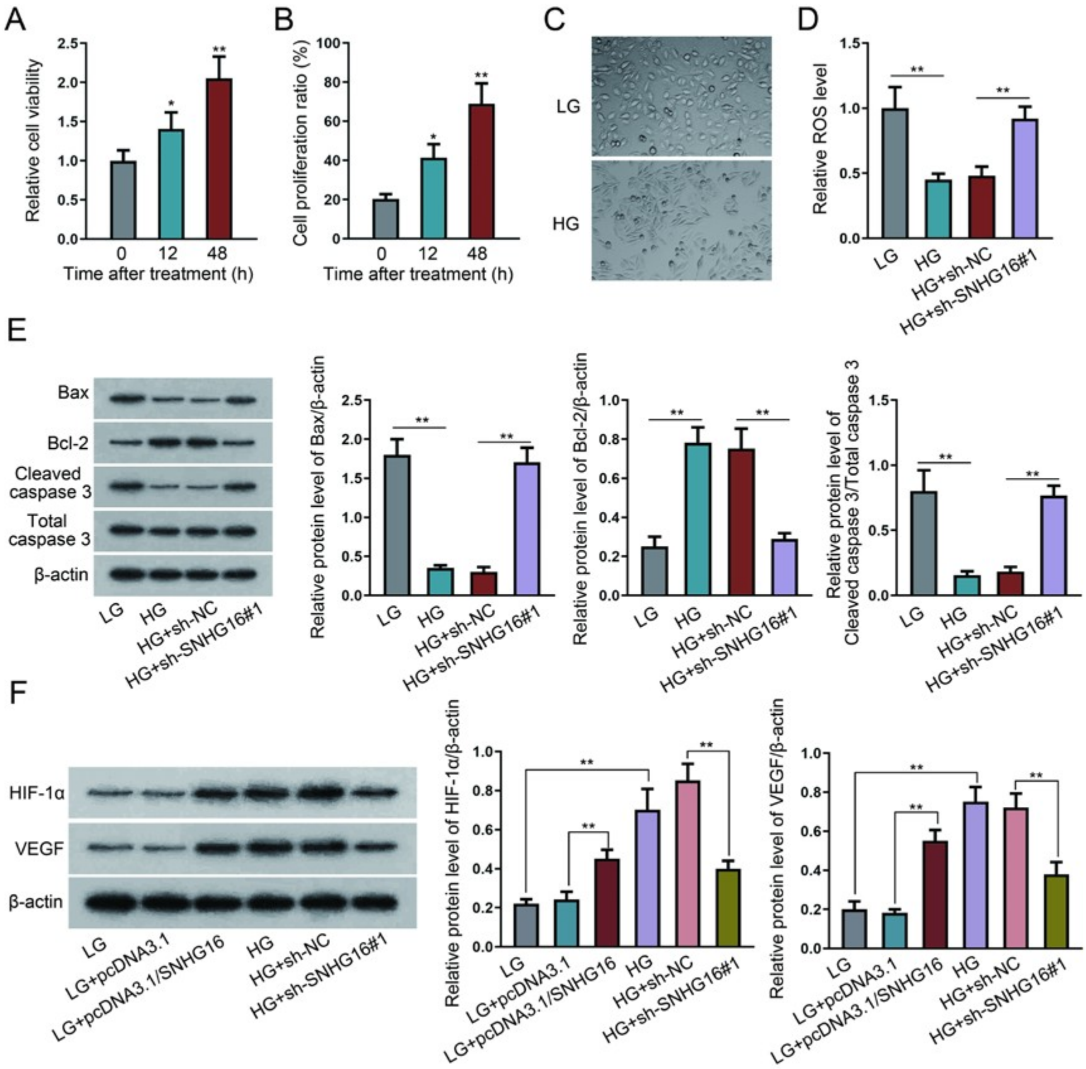
A
Relative cell viability
Time after treatment (h)
B
Cell proliferation ratio (%)
Time after treatment (h)
C
LG
HG
D
Relative ROS level
LG
HG
HG+sh-NC
HG+sh-SNHG16#1
E
Bax
Bcl-2
Cleaved caspase 3
Total caspase 3
β-actin
Relative protein level of Bax/β-actin
Relative protein level of Bcl-2/β-actin
Relative protein level of Cleaved caspase 3/Total caspase 3
F
HIF-1α
VEGF
β-actin
LG
LG+pcDNA3.1
LG+pcDNA3.1/SNHG16
HG
HG+sh-NC
HG+sh-SNHG16#1
Relative protein level of HIF-1α/β-actin
Relative protein level of VEGF/β-actin

Figure S2 The role of the snoRNAs encoded by SNHG16 in modulating hRMEC functions. (A) The expression level of different isoforms of SNHG16 in hRMECs cultured under HG or LG condition for 48 h was examined by qRT-PCR. N=3 in each group. (B) The expression levels of three snoRNAs were examined in hRMECs cultured under HG or LG condition for 48 h was examined by qRT-PCR. N=3 in each group. (C) The expression levels of three snoRNAs were examined in LG-induced hRMEC with SNHG16 overexpression or HG-induced hRMEC with SNHG16 silencing by qRT-PCR. N=3 in each group. (D) The cell viability was measured by CCK-8 assay after silencing of three snoRNAs. N=3 in each group. (E) EdU assay was applied to detect proliferative cells in HG-induced hRMEC after knockdown of three snoRNAs. N=3 in each group. (F) Wound healing assay revealed the snoRNAs silencing on hRMEC migration. N=3 in each group. (G) Migrated cell number of HG-induced hRMEC was counted using Transwell assay after silencing of snoRNAs. N=3 in each group. (H) qRT-PCR analysis of VEGF mRNA level in each group. N=3 in each group. (I) ELISA showed VEGF protein level influenced by snoRNAs knockdown. N=3 in each group. (J) The effect of snoRNAs knockdown on tube formation ability was evaluated by tube formation assay. N=3 in each group. All data were acquired from three independent experiments and presented as the mean ± SD.

**p < 0.01, n.s. indicated difference was not statistically significant.

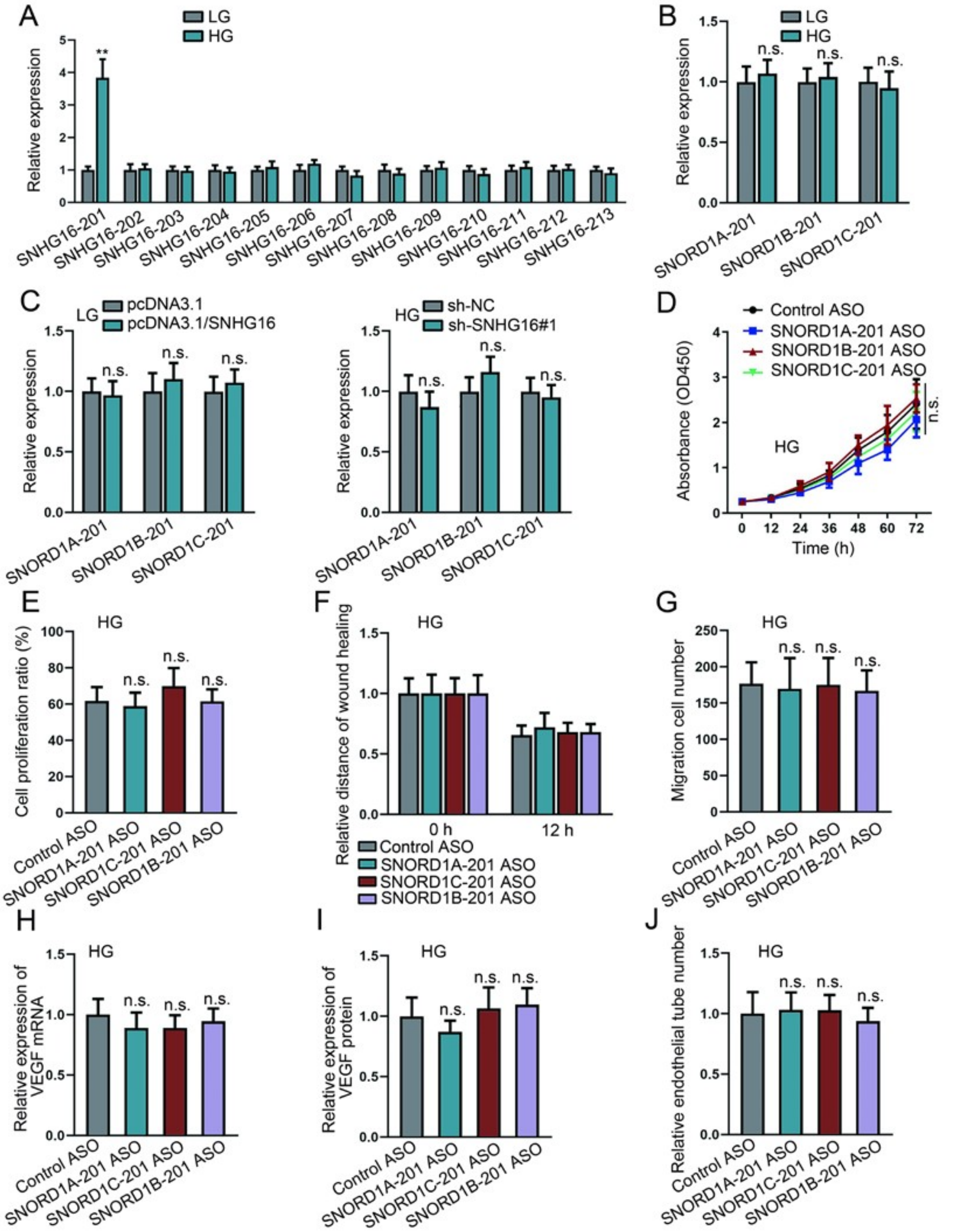
A
LG
HG
Relative expression
**
SNHG16-201
SNHG16-202
SNHG16-203
SNHG16-204
SNHG16-205
SNHG16-206
SNHG16-207
SNHG16-208
SNHG16-209
SNHG16-210
SNHG16-211
SNHG16-212
SNHG16-213
B
LG
HG
Relative expression
n.s.
SNORD1A-201
SNORD1B-201
SNORD1C-201
C
LG
pcDNA3.1
pcDNA3.1/SNHG16
HG
sh-NC
sh-SNHG16#1
Relative expression
n.s.
SNORD1A-201
SNORD1B-201
SNORD1C-201
D
Control ASO
SNORD1A-201 ASO
SNORD1B-201 ASO
SNORD1C-201 ASO
Absorbance (OD450)
HG
n.s.
Time (h)
E
HG
Cell proliferation ratio (%)
n.s.
Control ASO
SNORD1A-201 ASO
SNORD1C-201 ASO
SNORD1B-201 ASO
F
HG
Relative distance of wound healing
0 h
12 h
Control ASO
SNORD1A-201 ASO
SNORD1C-201 ASO
SNORD1B-201 ASO
G
HG
Migration cell number
n.s.
Control ASO
SNORD1A-201 ASO
SNORD1C-201 ASO
SNORD1B-201 ASO
H
HG
Relative expression of VEGF mRNA
n.s.
Control ASO
SNORD1A-201 ASO
SNORD1C-201 ASO
SNORD1B-201 ASO
I
HG
Relative expression of VEGF protein
n.s.
Control ASO
SNORD1A-201 ASO
SNORD1C-201 ASO
SNORD1B-201 ASO
J
HG
Relative endothelial tube number
n.s.
Control ASO
SNORD1A-201 ASO
SNORD1C-201 ASO
SNORD1B-201 ASO

Figure S3 The effect of SNHG16 on the expression of IRAK1 and IRS1. Experiments were conducted in cells treated with LG (5 mmol/L) or HG (25 mmol/L) for 48 hours. (A and B) IRAK1 and IRS1 expression in hRMECs cultured with HG, LG or osmotic control was detected using qRT-PCR and Western blot analyses. N=3 in each group. (C and D) qRT-PCR and Western blot analyses showed the mRNA and protein levels of IRAK1 and IRS1 influenced by SNHG16 overexpression or knockdown. N=3 in each group. All data were acquired from three independent experiments and presented as the mean ± SD. **p < 0.01, n.s. indicated difference was not statistically significant.

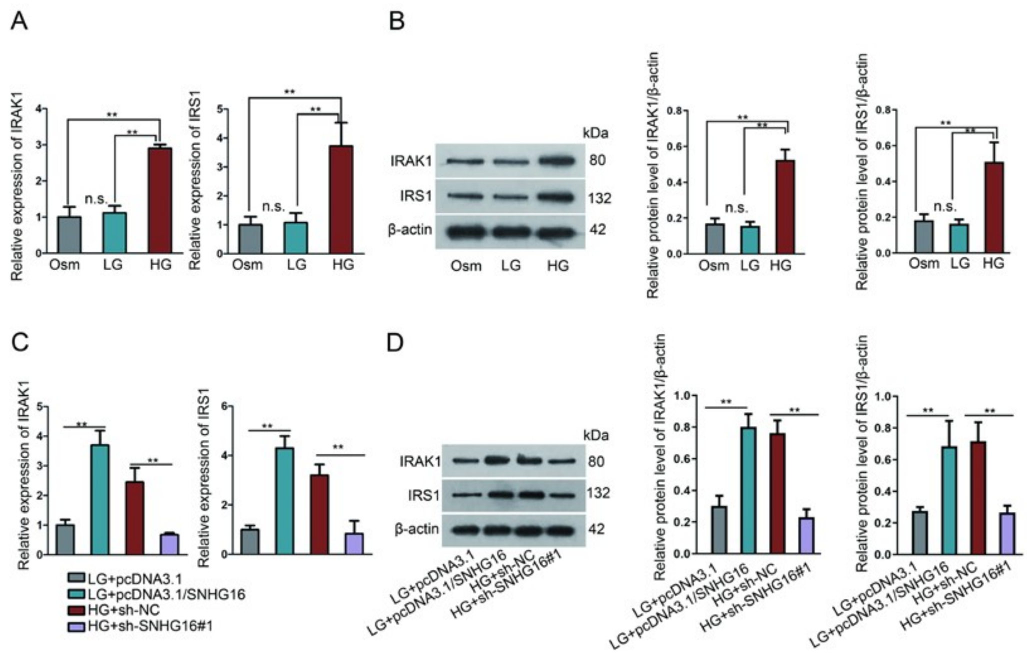

Figure S4 The effect of SNHG16 on NF-κB pathway activation through IRAK1. Experiments were conducted in cells treated with LG (5 mmol/L) for 48 hours. (A) Western blot analysis of IRAK1, p-IκBα and IκBα illustrated the change of IκBαphosphorylation level in response to IRAK1 knockdown or BAY 11-7082 treatment. N=3 in each group. (B) Western blot analysis of nuclear and cytoplasmic p65 illustrated the change of p65 nuclear translocation level in response to IRAK1 knockdown or BAY 11-7082 treatment. N=3 in each group. All data were acquired from three independent experiments and presented as the mean ± SD. **p < 0.01, n.s. indicated difference was not statistically significant.

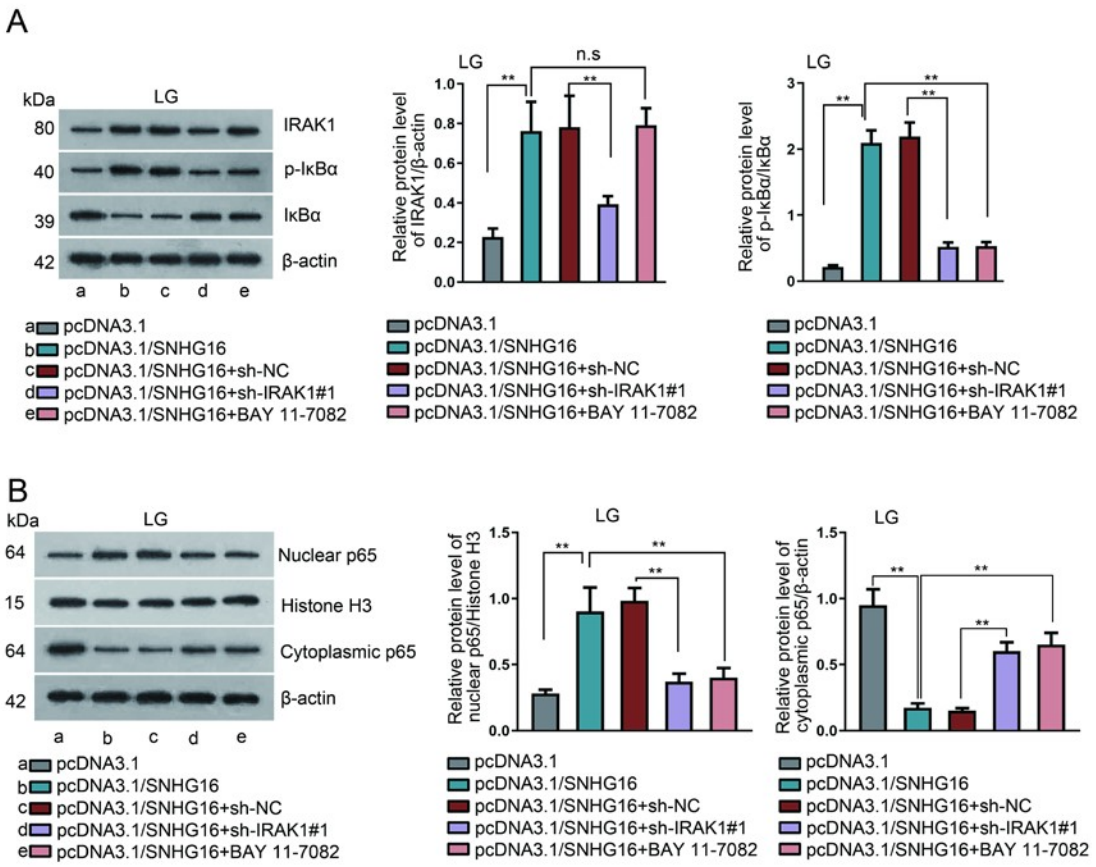

Figure S5 SNHG16 promotes hRMEC dysfunction via NF-κB pathway activation through IRAK1. Experiments were conducted in cells treated with LG (5 mmol/L) for 48 hours. (A and B) CCK-8 assay and EdU assay showed the effects of IRAK1 shRNAs and JSH-23 on cell proliferation in SNHG16-overexpressed hRMECs. N=3 in each group. (C and D) Wound healing assay and Transwell assay showed the effects of sh-IRAK1#1 transfection and JSH-23 addition on cell migration in hRMECs after SNHG16 overexpression. N=3 in each group. (E and F) qRT-PCR and ELISA showed the effects of sh-IRAK1#1 transfection and JSH-23 addition on VEGF level in hRMECs after SNHG16 overexpression. N=3 in each group. (G) Tube formation assay showed the effects of sh-IRAK1#1 transfection and JSH-23 addition on angiogenesis in SNHG16-overexpressed hRMECs. N=3 in each group. (H) ELISA-based NF-κB activity assay showed the change of NF-κB activity in response to IRAK1 knockdown or JSH-23 treatment. N=3 in each group. All data were acquired from three independent experiments and presented as the mean ± SD. $*p < 0.05$, $**p < 0.01$.

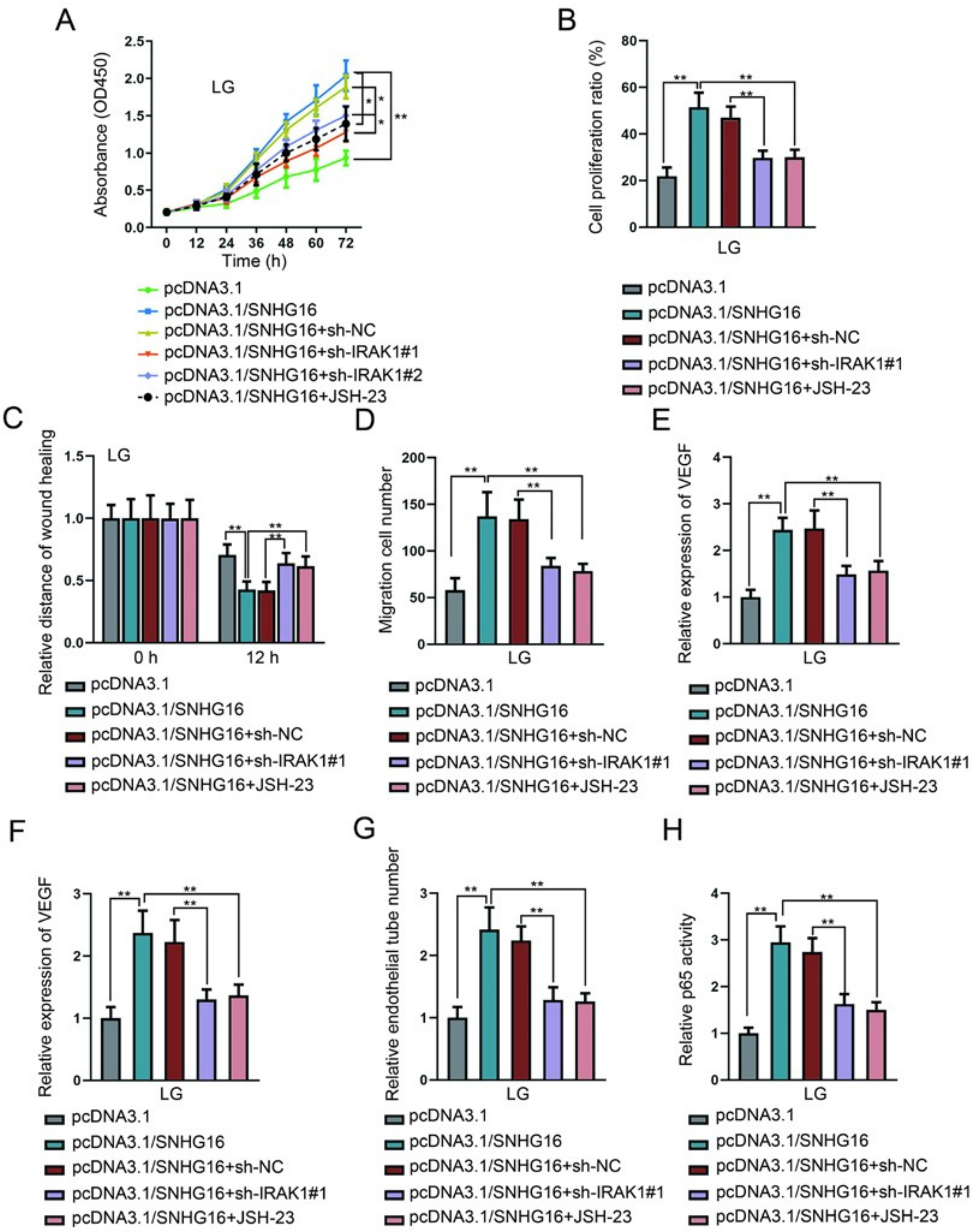
A
LG
Absorbance (OD450)
Time (h)
pcDNA3.1
pcDNA3.1/SNHG16
pcDNA3.1/SNHG16+sh-NC
pcDNA3.1/SNHG16+sh-IRAK1#1
pcDNA3.1/SNHG16+sh-IRAK1#2
pcDNA3.1/SNHG16+JSH-23
B
Cell proliferation ratio (%)
C
Relative distance of wound healing
0 h
12 h
D
Migration cell number
E
Relative expression of VEGF
F
Relative expression of VEGF
G
Relative endothelial tube number
H
Relative p65 activity

Figure S6 The effect of SNHG16 on NF-κB pathway activation. Experiments were conducted in cells treated with LG (5 mmol/L) for 48 hours. (A) Western blot analysis of IRAK1, p-IκBα and IκBα illustrated the change of IκBα phosphorylation level in response to IRAK1 knockdown or JSH-23 treatment. N=3 in each group. (B) Western blot analysis of nuclear and cytoplasmic p65 illustrated the change of p65 nuclear translocation level in response to IRAK1 knockdown or JSH-23 treatment. N=3 in each group. All data were acquired from three independent experiments and presented as the mean ± SD. **p < 0.01, n.s. indicated difference was not statistically significant.

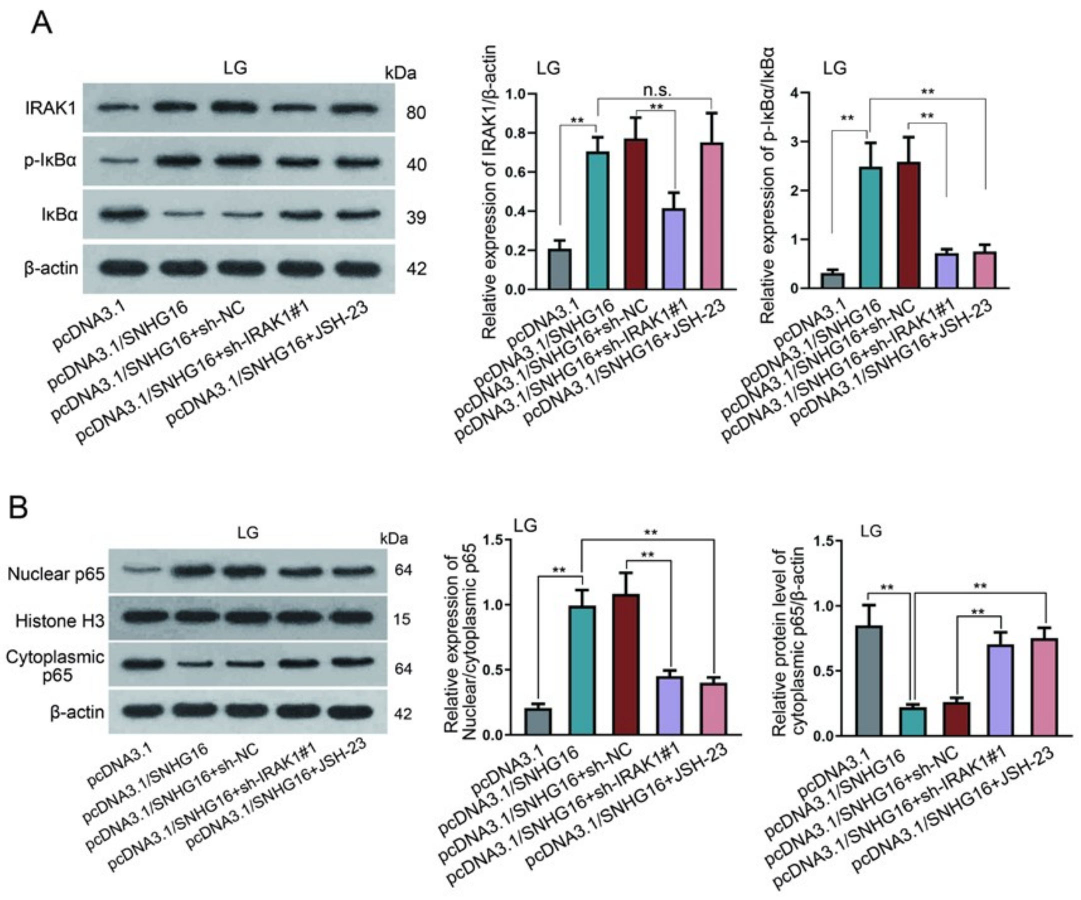

Figure S7 SNHG16 regulates PI3K/AKT pathway in LG-induced hRMECs. Experiments were conducted in cells treated with LG (5 mmol/L) for 48 hours. (A) Western blot analysis of IRS1, PI3K, p-AKT and AKT illustrated the change of PI3K/AKT pathway activity in response to IRS1 knockdown or LY294002 treatment. N=3 in each group. All data were acquired from three independent experiments and presented as the mean ± SD. **p < 0.01, n.s. indicated data was not statistically significant.

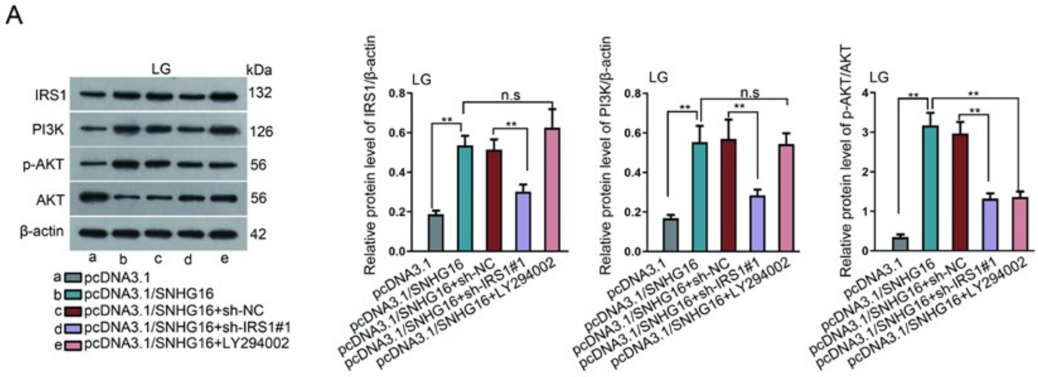

Figure S8 SNHG16 promotes hRMEC dysfunction via PI3K/AKT pathway activation through IRS1. Experiments were conducted in cells treated with LG (5 mmol/L) for 48 hours. (A and B) CCK-8 assay and EdU assay showed the effects of IRS1 shRNAs and ZSTK474 on cell proliferation in SNHG16-overexpressed hRMECs. N=3 in each group. (C and D) Wound healing assay and Transwell assay showed the effects of sh-IRS1#1 transfection and ZSTK474 addition on cell migration in hRMECs after SNHG16 overexpression. N=3 in each group. (E and F) qRT-PCR and ELISA showed the effects of sh-IRS1#1 transfection and ZSTK474 addition on VEGF level in hRMECs after SNHG16 overexpression. N=3 in each group. (H) Tube formation assay showed the effects of sh-IRS1#1 transfection and ZSTK474 addition on angiogenesis in SNHG16-overexpressed hRMECs. N=3 in each group. All data were acquired from three independent experiments and presented as the mean ± SD.

*$p < 0.05$, **$p < 0.01$.

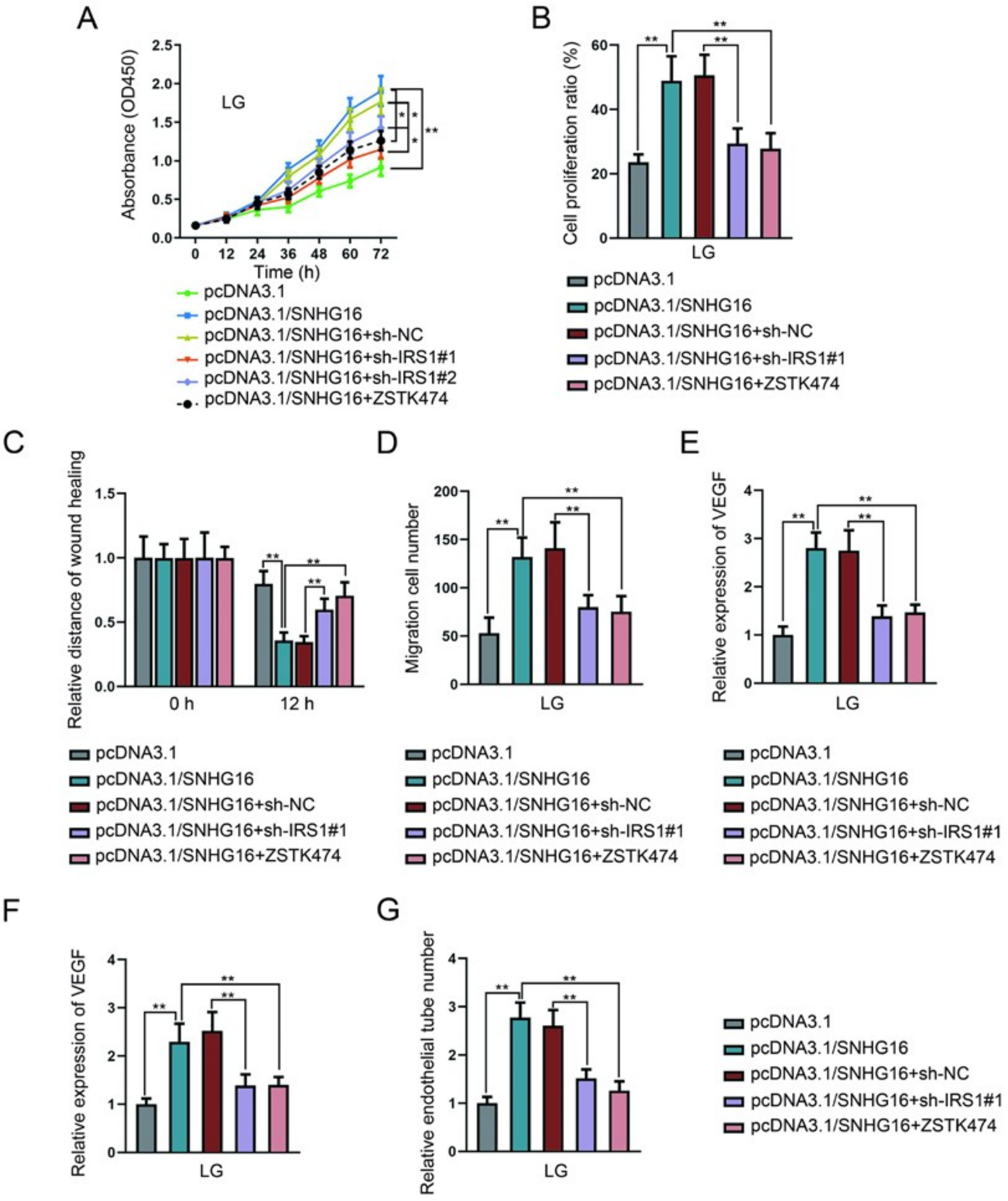
A
LG
Absorbance (OD450)
Time (h)
pcDNA3.1
pcDNA3.1/SNHG16
pcDNA3.1/SNHG16+sh-NC
pcDNA3.1/SNHG16+sh-IRS1#1
pcDNA3.1/SNHG16+sh-IRS1#2
pcDNA3.1/SNHG16+ZSTK474
B
Cell proliferation ratio (%)
LG
C
Relative distance of wound healing
0 h
12 h
D
Migration cell number
E
Relative expression of VEGF
F
Relative expression of VEGF
G
Relative endothelial tube number

Figure S9 SNHG16 regulates PI3K/AKT pathway in LG-induced hRMECs. Experiments were conducted in cells treated with LG (5 mmol/L) for 48 hours. (A) Western blot analysis of IRS1, PI3K, p-AKT and AKT illustrated the change of PI3K/AKT pathway activity in response to IRS1 knockdown or ZSK474 treatment. N=3 in each group. All data were acquired from three independent experiments and presented as the mean ± SD. **p < 0.01, n.s. indicated difference was not statistically significant.

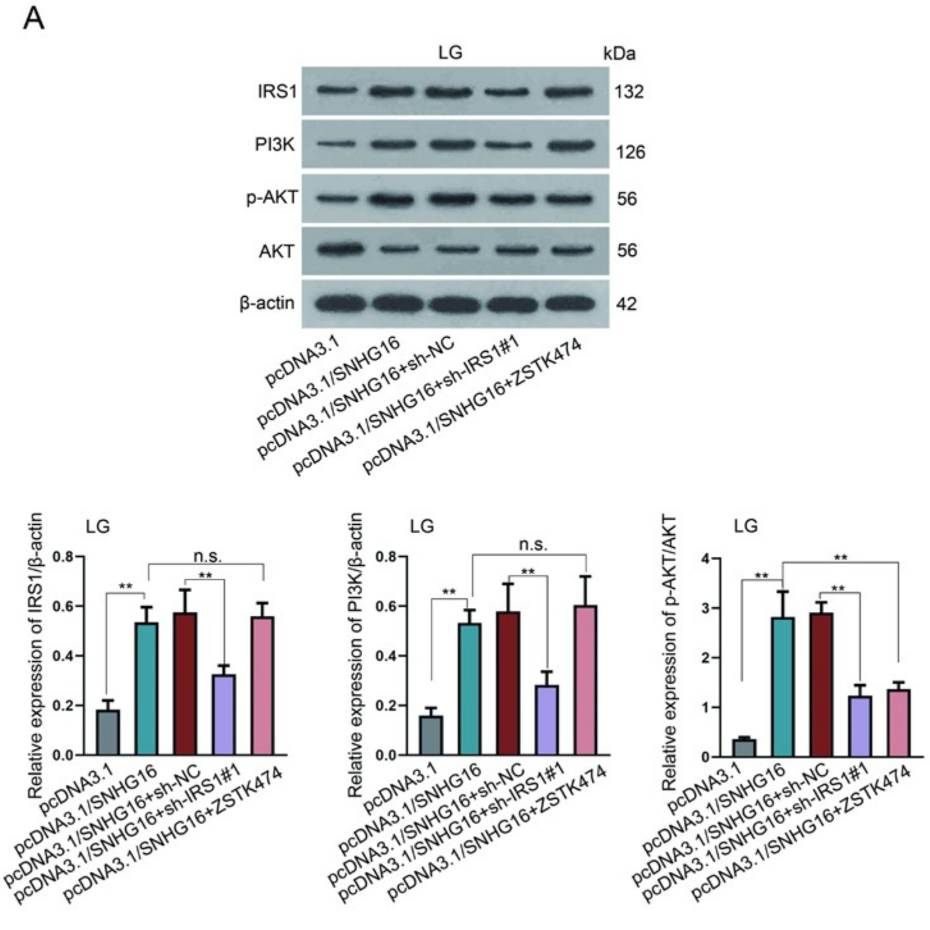

Table S1 qRT-PCR primers.

Gene ID	Primer	Sequence (5′-3′)
SNHG16	Forward	GCTGTGGCCTTGAAAACAGTC
	Reverse	GGGAGCTAACTCACATTAAAGACA
VEGF	Forward	AAGGAGGAGGGCAGAATCAT
	Reverse	ATCTGCATGGTGATGTTGGA
miR-146a-5p	RT	CTCAACTGGTGTCGTGGAGTCGGCAATTCAGTTGAGCAACCCA
	Forward	GCCGAGTGAGAACTGAATTCCA
miR-7-5p	RT	GTCGTATCCAGTGCAGGGTCCGAGGTATTCGCACTGGATACGACAAC
	Forward	CGCTGGAAGACTAGTGATTTT
Universal Primer	Reverse	CTCAACTGGTGTCGTGGA
IRAK1	Forward	TCAGAACGGCTTCTACTGCCTG
	Reverse	TACCCAGAAGGATGTCCAGTCG
IRS1	Forward	CTTCTCAGACGTGCGCAAGG
	Reverse	GTTGATGTTGAAACAGCTCTC
CARD10	Forward	CTTCGTCCTCATCCTCTGACAG
	Reverse	AGACACCCGAATAGCCAGAGAC
TRAF6	Forward	CAATGCCAGCGTCCCTTCCAAA
	Reverse	CCAAAGGACAGTTCTGGTCATGG
GAPDH	Forward	GGAGCGAGATCCCTCCAAAAT
	Reverse	GGCTGTTGTCATACTTCTCATGG
U6	Forward	CTCGCTTCGGCAGCACA
	Reverse	AACGCTTCACGAATTTGCGT

Table S2 shRNA sequences and FISH probe sequence.
sh-NC:
CCGGTGAATCTCAACTACATGTTTGCTCGAGCAAACATGTAGTTGAGATTCA
TTTTTG
sh-SNHG16#1:
CCGGACATTTTCTCAACGTTTGGAACTCGAGTTCCAAACGTTGAGAAAATG
TTTTTG
sh-SNHG16#2:
CCGGATCTGTAAAGCTAAATATGAACTCGAGTTCATATTTAGCTTTACAGAT
TTTTG
sh-SNHG16#3:
CCGGACAAAAGATACCATTTCAGACCTCGAGGTCTGAAATGGTATCTTTT
sh-NC:
CCGGGTGATGGTGATGAGTAGTCTACTCGAGTAGACTACTCATCACCATCA
CTTTTTG
sh-IRS1#1:
CCGGGGGTTTGGAGAATGGTCTTAACTCGAGTTAAGACCATTCTCCAAACC
CTTTTTG
sh-IRS1#2:
GTACCGGACTCATTGCCAAGATCCTTTACTCGAGTAAAGGATCTTGGCAAT
GAGTTTTTTTG
sh-IRS1#3:
CCGGGCCGCTCAAGTGAGGATTTAACTCGAGTTAAATCCTCACTTGAGCGG
CTTTTTG
sh-NC:
CCGGACGTGATAGAGTACTGATCTACTCGAGTAGATCAGTACTCTATCACGT
TTTTTG
sh-IRAK1#1:
CCGGTGGTGAAACAGAGCTTCTTAACTCGAGTTAAGAAGCTCTGTTTCACC
ATTTTTG
sh-IRAK1#2:
CCGGCAATTGCTGCCCAGATCTATACTCGAGTATAGATCTGGGCAGCAATTG
TTTTTG
sh-IRAK1#3:
CCGGTACCGAGCAGTCATGAGAAATCTCGAGATTTCTCATGACTGCTCGGT
ATTTTTG

Sequence for SNHG16-probe used in FISH assay
ugacgguaguuucccaaguuuauuguaagugguuuuaaguuaagucucauccaaacaaguuaucacacagcac
uuaaccaagcccugggauuuacugucuugaugacuacacggcuuugcacagucugagaugcuucagugugcaa
ggcagcagcuggggggggaggagggggucuucacagggacagcuggcaagagacuuccugaggcacaucagu
uacguuggucaauuuagggcacggucugguucugcagcuuugaaagguggauucuuucuauuagcacacuuu
acaagagggauuguaaaggauuaacucagucaccagaaacgaaacaccacuucagaaauucagagaccucugau
caacagaacagacauuugggcuuuaacugcuaaagcagcuaccuacuuggggaaaccauggcauucugcugcc

uggacagcaggaauuaagagagauuucagaguuacuggcacgaggacaaagccucucagcucgcuucaccuug
gcaaccuuaaacagucccuccuuggucucuaugauagcaguauguuccuuuggauggaauacuugguuugga
aauaauguagucaaauguaggauauguaaguacuccucaacagucacaauccauccuaguaucuuaaauagug
auuuuuuuuuuuaauuuacaaaagagguuuauuggacuuacaguucuacauggcugggaaggccucacaau
cauggcggaaggugaaagccacaucucacauggcagcagauaagagaaaaaagguagugaucuuuuaguaaa
gaaaccugagacugguagugggcuuggagccagaggaucgcuuaaguccgggaguucgagaccagccagggu
aacaaauugagacccccccccaacuuuaaaauuaaaaaacgaaagaaaaaauagcuggguguggugcucacac
cuguaauccuagcacuuugggaggccaaggcggguggauugccugagcucaggaguuugagaccaaccuggg
caacaugguugaaacccugucucuacuaaaacaaaaaaaaucagccagguggugguagcgugcaccuguaguccc
aguuacuugggaggcugaggcacaagaauugcuugaaccugggagguagagguugcagagaucaugccacug
cacucccagccugggugacagagcaagacugucucaaaaacaacaacaacaacaacaaaacaaaacaaaaaaa
accucucaaaaaaaugaaaaaaaauuuaaauuuaaauuaaaaaaaaaaaugcugggucugauggcucacaccug
ucaucucagcacuuugggaagcugaggcaggcagaucacaaggucaggagauagagaccauccuggccaacau
ggugaaacccagucucuacuaaagaagguacgcgccuguagucccagcacucaggaggcugagccaggagaau
ugcuugaacccgggagguggagguugcagugagccaagauuguaccacuucacuccagccuggcgacagagug
gaacuccgucucaaaaaauaaaauaaaauaaaauaaaauaaaauaaaauaaaauaaaauaaagcaaaaauauaaa
auguuaaaaaaaaaacaaaaaaagggaaaaaggaagcugauugccuuggugagucaacacugggguauuuucug
accacuauuugaaacaaaaaaggaaaccacugauauucuaugcaaagaucuguuccuggaaggcacucugcgga
gacaccaggagaacuuuuaucaauccuucauugauuugaaguaaaagugcuaaagcaaugguuguggguggca
acccauuagcagaucacaaaaucacuguagugguaacuaaacaagaggaaacacaagacggcauccuguguaa
cugggguuaagcauuacucucugaaacucauggcaucaguuuccucuuaggcucuuccaacagaguauaauca
uguucauuucaguuuacaauccuugcagucccaucgauuugugagauuauacccaggucauccacaguggagg
ucugaaaauguucuuaguuguacucuaauuucacuaacugccaaaagguuuuccagaauaaucucaguugcuu
cauuccuuuaaagaugaagcccaaagaacgcauggcgauuacuuuagaggaacaauuagcagcagaggcaggg
cugugcugaucccaucuggcaucgcugggagcuaacauuaaagacauggcacuuuggguccggguccagguccc
ugguucagagcagcugccacaccguggcuacuagaggauccuuuuccggcuuuggaaacugaggcugacugca
ccauuuacagaaauuucaucaugaagauccuuacacuuaacaccuuaaggugaugaguuuucacaugacuguu
uucaaggccacagccaccaccuuuaaucuaaaagagaaggauaacaggcuucauucuaacagaaaccaccuuaa
gguuuauucuccagauuuguauuaaacagcugagccucagcaagucagaccuccucacaaaagauaccauuuc
agacagggauaacuuuaaaucauccucagcucagccaauaacaaagauuccuaaauccuagcgauccaaaauuc
auuugccaugucucuuucaucccugcaugcccuaacgaggauccacgcugguuacaaugacaccuaguggccc
caaaucacacgggcaaagacgcuacucauuuuuaucauuaaaauaauuagcauucugaauuuugaguaauuuc
uuaccauagcuggcuuguaugucaacacguauucucauacuugaacuucagaauaaagcaggaggugaaugua
ugugauggcaccaacugugcuuucucauuucuagugaccacucaggcccugccugcacccacucuuacucaug
uuaagggcuccaucuauuacucugauccuucaaccuggagcucugcuuuucaccgucucugcagaugccaaac
aaagaaaggccucugggggaggaaaaaaggcucguuguuaaaacuacggcuuuccagaaguuuacaauccuau
ugacuccuaucuguaaagcuaaauaugaacuaccgauuggggccguccucaagacacuccaagcggaaucacg
ucuucacacguuaacacuccugaugcugaauuaagaaccaccaucgucagagucugcccgugcgcgggcaccg
aagaccccugggcaugacgcucagcacacgcgcggcggaggcgcacggggcugcccagguuguuaggcgcucu
cccccgcagaagccggcccuaugacgccgaggacgggcuugagcucucucccuucacauaguaacauuuucuc
aacguuuggaaagcauuuucucauccaguacuuuuaacccuccuaaccacccuaugagggagaaaccgagauu
aucgucaccacuucgucucugaggaagggaagccaaguauguuagugucgugucaaggaagaccccguuuucc
cacccaggcagccuuaccaucaucacuaaaggccugagacugcucgccgugcucaacaccgacuggaguggcca
ugucuuccagccacgcggccgac
